# Ruthenium^II^ Complexes bearing Fused Polycyclic Ligands: From Fundamental Aspects to Potential Applications

**DOI:** 10.3390/molecules19045028

**Published:** 2014-04-22

**Authors:** Ludovic Troian-Gautier, Cécile Moucheron

**Affiliations:** Laboratoire de Chimie Organique et Photochimie, Université Libre de Bruxelles (ULB), CP160/08, 50 av. F. D. Roosevelt, 1050 Bruxelles, Belgium; E-Mail: ltroiang@ulb.ac.be

**Keywords:** ruthenium^II^, polypyridyl, polyazaaromatic, extended π-system, photo-catalysis, photophysics

## Abstract

In this review, we first discuss the photophysics reported in the literature for mononuclear ruthenium complexes bearing ligands with extended aromaticity such as dipyrido[3,2-a:2',3'-c]phenazine (DPPZ), tetrapyrido[3,2-a:2',3'-c:3'',2''-h:2''',3'''-j]-phenazine (TPPHZ), tetrapyrido[3,2-a:2',3'-c:3'',2''-h:2''',3'''-j]acridine (TPAC), 1,10-phenanthrolino[5,6-b]1,4,5,8,9,12-hexaazatriphenylene (PHEHAT) 9,11,20,22-tetraaza- tetrapyrido[3,2-a:2',3'-c:3'',2''-l:2''',3'''-n]pentacene (TATPP), *etc.* Photophysical properties of binuclear and polynuclear complexes based on these extended ligands are then reported. We finally develop the use of binuclear complexes with extended π-systems for applications such as photocatalysis.

## 1. Introduction

Considerable attention has been paid during the last decades to the study of polyazaaromatic transition metal complexes as DNA photoprobes or photoreagents [1,2,3,4,5,6,7,8]. The interest in such coordination compounds originates both from their luminescence and photoreactive properties with DNA and from their particular coordination geometry. The three-dimensional shapes of these rigid complexes make them excellent candidates to recognise complementary shapes in different DNA binding sites. The interaction with the chiral nucleic acids may be enantioselective when the complexes are chiral themselves. The extensively studied [Ru(phen)_3_]^2+^ has been the first substitution inert complex for which selective binding of the ∆ enantiomer to B-DNA has been reported (enantioselectivity in binding of metal complexes to DNA is presented in [9]). From these initial studies, a research area rapidly focused on the development of new DNA binding complexes.

The luminescence and photoreactivity of those compounds with nucleic acids allow, among other things, to mark reversibly or irreversibly the DNA at the sites of interaction of the complexes. Thus for example, Ru^II^ complexes have been used as luminescent reporters for DNA instead of using a radioactive label [10,11,12]. Certain metal complexes can also induce photocleavages of the DNA backbone and can be regarded as artificial endonucleases [13,14,15,16]. Other complexes can produce DNA photoadducts and can then play the role of irreversible DNA markers or act as photo-reagents in view of phototherapeutical applications [17,18,19,20]. The formation of photoadducts of Ru^II^ complexes on DNA can indeed interfere with the normal functions of DNA and inhibit for example the RNA polymerase.

It has been shown that the metallic complexes interact with DNA according to different geometries [9,21,22] among which intercalative binding deserved extra interest. The first ligand designed to form metallo-intercalators is the extended aromatic compound dipyrido[3,2-a:2',3'-c]phenazine (DPPZ) [23,24], whose synthesis was described in 1970 [25]. Complexes based on DPPZ have been extensively studied due to their strong intercalation in the DNA double helix and their “light switch” effect [26,27,28,29,30]. Indeed, whereas [Ru(bpy/phen)_2_(DPPZ)]^2+^ (bpy = 2,2'-bipyridine, phen = 1,10-phenanthroline) is non emissive in water at room temperature, it emits brightly upon addition of DNA. Since this discovery numerous related DNA “light switch” complexes, such as DPPZ analogues or TPPHZ complexes [31], have been reported in the literature and used as photoprobes or reagents for biomolecules (see below).

The DPPZ complexes and their DNA binding have been extensively studied, using numerous advanced techniques such as linear dichroism [29], picosecond time-resolved spectroscopies [32,33,34,35], resonance Raman [36,37,38] or, very recently, X-ray crystallography [39,40,41,42]. DPPZ complexes have been used as probes for liposome membranes [43,44] or for staining nuclear components [45]. They also found applications in the development of sensors and in the host-guest design [46,47,48,49,50,51,52]. 

Binding geometry and photophysical properties of semi-flexible binuclear ruthenium complexes based on two DPPZ ligands connected with a single bond have also been reported [53,54,55]. 

As it will be discussed in more detail below, the investigation of the electronic and photophysical properties of [Ru(bpy/phen)_2_(DPPZ)]^2+^ have drawn considerable attention in order to understand the origin of the “light switch” effect in those complexes.

At the same time, extended ligands have also been used in a quite different area. Indeed other ligands have been designed, which are based on the DPPZ moiety or other planar ligands but present the extra ability of playing the role of bridging ligand, thanks to the presence of two, three or more chelation sites. Thus binuclear complexes, dendrimers or edifices of higher nuclearity, homo- or heterometallic compounds, based on rigid heteroaromatic ligands have been constructed for applications in light-harvesting [56,57,58,59,60], energy transfer and artificial photosynthesis [61,62,63,64,65], potential photoconversion devices [66], non-linear optics [67,68], opto-electronics or photocatalysis [69,70,71], as they exhibit a rich electrochemistry and photophysics.

Of course a deep understanding of the electronic and photophysical properties of those polynuclear complexes is necessary in order to design edifices with the expected properties for a specific application.

This review will be focused on the photophysical and photochemical properties of polyazaromatic trischelated Ru^II^ complexes bearing DPPZ derivatives and other extended aromatic ligands. Behaviors of the mononuclear complexes with DNA will not be discussed here as they are already reported elsewhere [4,9,20]. The electronic and photophysical properties of the mononuclear complexes will be discussed and compared as a prerequisite before discussing the properties of complexes of higher nuclearity based on the same planar ligands. The last part of this review will overview more applied systems based on extended planar ligands and developed for photocatalysis applications.

## 2. Mononuclear Ruthenium^II^ Complexes

### 2.1. [Ru(bpy)_3_]^2+^

**Figure 1:**
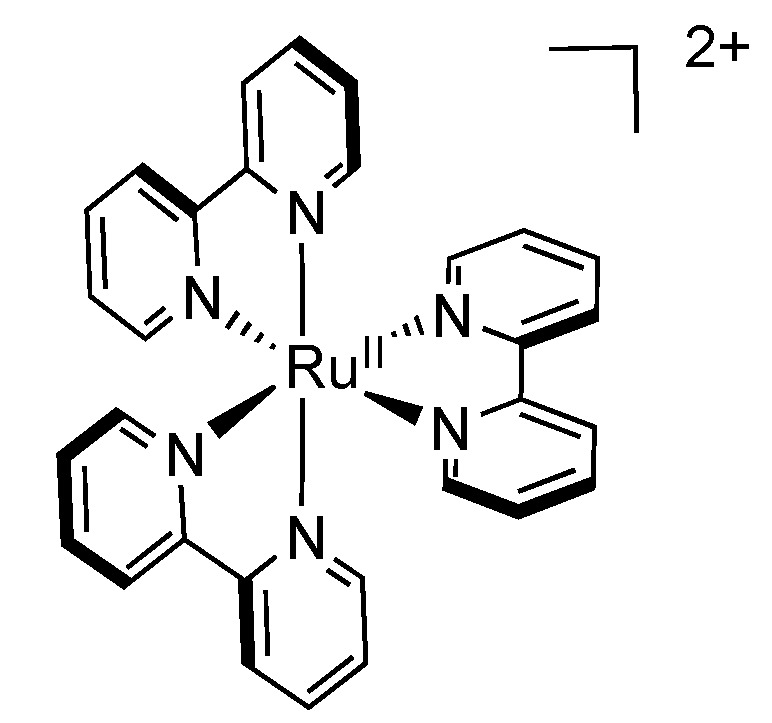
[Ru(bpy)_3_]^2+^_._

The synthesis of [Ru(bpy)_3_]^2+^ (Figure 1) was first reported by Burstall in 1936 [72], but its luminescence was only observed in 1959 [73]. 

**Figure 2:**
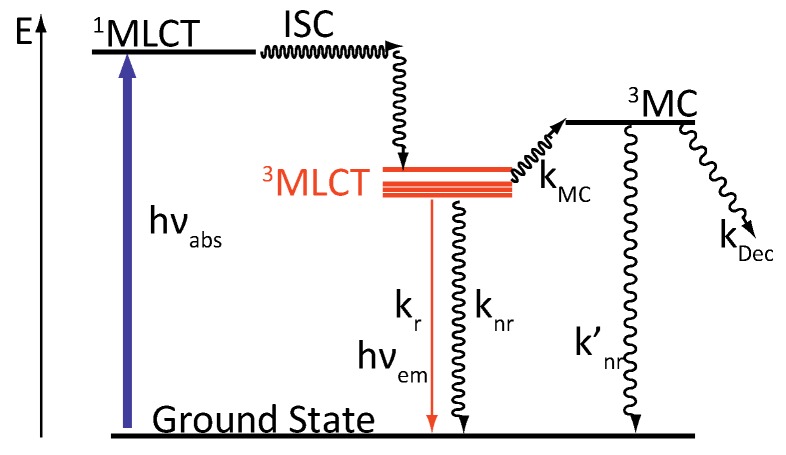
Photophysical scheme of [Ru(bpy)_3_]^2+^.

Since then, extensive studies have been performed on [Ru(bpy)_3_]^2+^, allowing the development of the generally accepted photophysical scheme for classical complexes as the following [74,75,76,77,78,79,80,81,82,83,84,85,86,87,88,89,90,91,92] (Figure 2). Absorption of a photon allows the population of a singlet metal to ligand charge transfer ^1^MLCT that has a lifetime of 40 ± 15 fs [93]. Due to the heavy metal center, an intersystem crossing ISC that mixes singlet and triplet states, allows the population of the ^3^MLCT state, localized on a single ligand. This ISC is completed in approximately 75 fs [81] and the complex reaches a “Thermally equilibrated excited state” THEXI-^3^MLCT. This ^3^MLCT excited state of [Ru(bpy)_3_]^2+^ can therefore be regarded as [Ru(III)(bpy)_2_(bpy•-)]^2+^*. Studies by Crosby *et al.* have revealed that this ^3^MLCT state is in fact composed of three low-lying MLCT states in rapid equilibrium that are separated by 10–90 cm^−1^. These states can only be distinguished at 77 K. Von Zelewsky and Meyer have later identified that a fourth MLCT with greater singlet character was also present at several hundred cm^−1^ to higher energy.


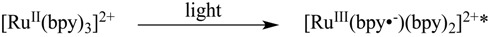


Once this ^3^MLCT state is populated, various deactivation processes can take place. First, a radiative decay that dissipates energy as a photon, brings the complex back to its ground state. This decay is responsible for the characteristic luminescence of numerous ruthenium complexes and is generally described by a radiative constant (k_r_). Second, a non radiative decay, characterized by the constant (k_nr_), brings the complex back to its ground state by dissipation of energy, principally as heat. Finally, a third process, called thermal activation, is needed to explain the lifetime dependence with temperature. This thermal activation leads to the population of a triplet metal center (^3^MC) state. Populating this state lowers the metal-ligand bond and can therefore induce the loss of a ligand, controlled by the dechelation constant (k_Dec_). Based on the different deactivation pathways, the following equation describing the excited state lifetime in function of temperature has been proposed:

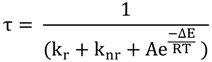

where ΔE represents the energy difference between the ^3^MLCT state and the ^3^MC state. This photophysical scheme is generally accepted as the model for all homoleptic complexes and for several heteroleptic complexes (Figure 2). 

As explained previously, the deactivation pathway for the excited state of [Ru(bpy)_3_]^2+^ is governed by two constants, k_r_ and k_nr_ and by thermal activation leading to the ^3^MC state. Solvent effects can of course influence these constants. After the Franck-Condon vertical transition from the ground state to the excited state, the dipole moment of the excited [Ru(bpy)_3_]^2+^ is bigger than in the ground state. Solvent molecules around the complex tend thus to reorganize in order to stabilize the ^3^MLCT excited state. Solvents with high polarity will therefore stabilize the excited state complex, meaning that the emission energy will be shifted to lower energy, corresponding to a red-shift in emission. Furthermore, it has been shown that k_r_ is only slightly solvent dependent but that k_nr_ values tend to increase while the emission energy decreases as expected for radiationless transitions according to the energy gap law. Parameters that are the most influenced by solvent are the terms included in the temperature dependent deactivation pathway, *i.e.*, the pre-exponential factor A, and ∆E. In polar solvents, the ^3^MLCT is stabilized, and therefore the energy between the ^3^MLCT and the ^3^MC tends to increase. On the contrary, when less polar solvents are used, the ^3^MLCT excited state is less stabilized and the energy difference between the ^3^MLCT state and the ^3^MC state tends to decrease, facilitating therefore the thermal activation towards this ^3^MC state. 

### 2.2. [Ru(bpy/phen)_2_(DPPZ)]^2+^

**Figure 3:**
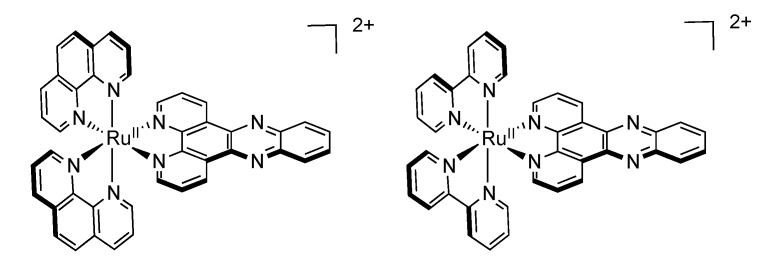
[Ru(phen)_2_(DPPZ)]^2+^ (left) and [Ru(bpy)_2_(DPPZ)]^2+^ (right).

For some complexes, the photophysical scheme can be different from the “classical” one. This is the case for ruthenium complexes containing the extended ligand DPPZ, such as [Ru(bpy/phen)_2_(DPPZ)]^2+^ (Figure 3). These two complexes exhibit a behavior that could not be explained by the classical photophysical scheme, namely the “light-switch” effect [24].

**Figure 4:**
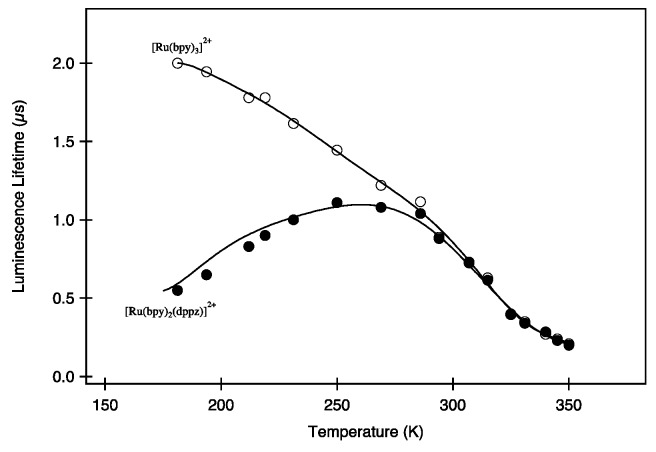
Schematic representation of the luminescence lifetime with temperature for [Ru(bpy)_3_]^2+^ and [Ru(bpy)_2_(DPPZ)]^2+^ [97].

This “light-switch” controls the luminescence properties of the complex as it is luminescent in organic solvents, but its luminescence is switched off in aqueous solutions. It was proposed that this “light switch off” is a consequence of the quenching induced by proton transfer, reducing therefore its emission quantum yield [24,94,95]. Nonetheless, it was necessary to wait until 1997 for a new photophysical scheme to be proposed by Barbara and coworkers [96]. This scheme introduced a supplementary MLCT state (MLCT'') whose implication in the deactivation process is very sensitive towards the direct environment of the complex, such as solvent. Later on, another model was elaborated after the studies of Papanikolas, Meyer *et al.* on [Ru(bpy)_2_(DPPZ)]^2+^. They performed an ingenious experiment, measuring the lifetime in butyronitrile as a function of temperature [97,98], ranging from 170 to 360 K (Figure 4). As previously explained, for classical complexes such as [Ru(bpy)_3_]^2+^, the lifetime continuously increases with lowering temperature. For [Ru(bpy)_2_(DPPZ)]^2+^ nonetheless, a maximum is observed in the same temperature range. This behavior could not be explained by the classical photophysical scheme, nor by the model proposed in 1997. This decrease in luminescence lifetime could only be explained by the presence of a non luminescent state, *i.e.*, a “Dark State” whose energy is lower than that of the luminescent one, meaning that its population is directly controlled by temperature. 

A new photophysical scheme was therefore published, which includes the following:

- A ^3^MC state higher in energy
- A luminescent ^3^MLCT “bright state” corresponding to a charge transfer from the ruthenium center to the 1,10-phenanthroline moiety of the DPPZ ligand.
- A “dark state”, non-luminescent and lower in energy, that corresponds to the phenazine moiety of DPPZ.

**Figure 5:**
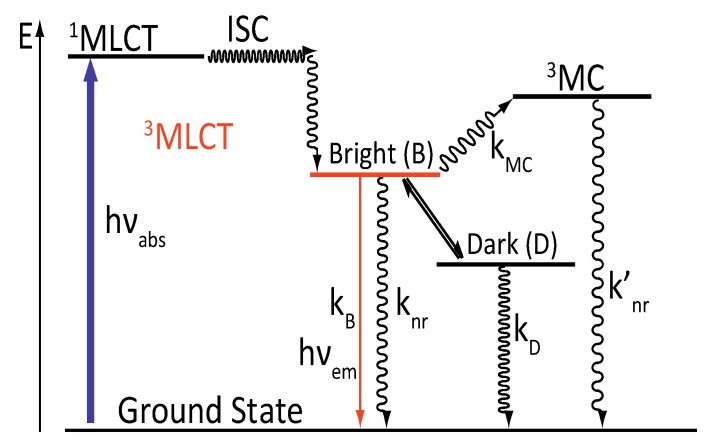
Photophysical scheme of [Ru(bpy/phen)_2_(DPPZ)]^2+^ in water.

This model offers a dynamic equilibrium between a luminescent state and a dark state, lower in energy (Figure 5). 

This new model proposed a new deactivation equation that can be correlated to the population-weighted average of the deactivation constant of the two states, bright (B) and dark (D):

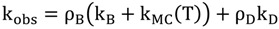

ρ_B_ and ρ_D_ correspond to the relative populations of the bright and dark states, respectively, populations that vary with the temperature. k_B_ and k_D_ are the kinetic relaxations towards the ground state while k_MC_ is the kinetic constant for activation towards the ^3^MC state. 

Analyzing the data allowed to extrapolate that the equilibrium is completely displaced towards the luminescent bright state when the temperature reaches 260K. The relative population of these two states is governed by an equilibrium constant:

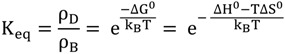


∆H° corresponds to the enthalpic change for the bright to dark process and ∆S° is the entropic change for the same process. The dark state is therefore favored enthalpically (∆H° < 0), meaning that when the temperature is low, the dark state will be mostly populated (K_eq_ >> 1), while at high temperature, the luminescent state will be populated, favored by a gain in entropy (K_eq_ << 1). 

**Figure 6:**
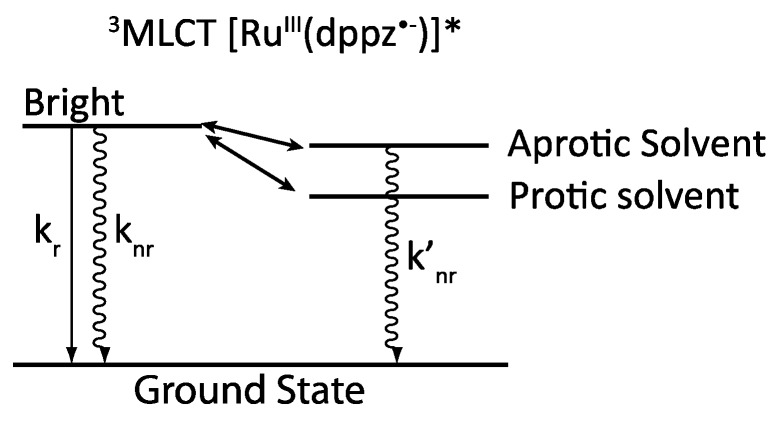
Solvent influence on the dark and bright states.

Observations made to develop the photophysical scheme proposed by Barbara in 1997 can also be included in this new model, as the different states that are involved in the photophysical scheme can be stabilized by different solvents. A protic solvent such as water will stabilize the dark state by hydrogen bonding. The dark state will therefore be lower in energy than the bright state, thus mainly populated at room temperature, causing the loss in emission. When an aprotic solvent is used, the energy of the dark state is less stabilized than in a protic solvent, and the bright state becomes accessible at room temperature (Figure 6).

**Figure 7:**
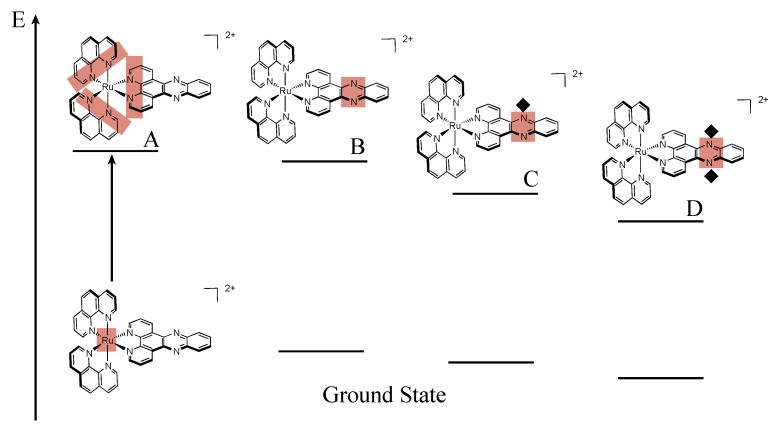
Schematic relaxation pathway for [Ru(phen)_2_(DPPZ)]^2+^ in polyol solvents [100].

Refinement of the model in polyol solvents suggests that [Ru(phen)_2_(DPPZ)]^2+^ exhibits three distinct excited states, that differ primarily by solvent interactions on the phenazine moiety [32,99,100], two of them being luminescent, and a third one being a dark state (Figure 7). Indeed, photo-excitation populates a precursor state [33] (Figure 7A) that relaxes towards an excited state localized on the DPPZ ligand, where no hydrogen bonds occur (Figure 7B). This excited state decays then rapidly towards an excited state where one of the nitrogens of the phenazine core is hydrogen-bonded to solvent (Figure 7C). This state is in equilibrium with a last excited state, the dark state, where both nitrogens are hydrogen-bonded to solvent (Figure 7D). It is important to note that the two models are complementary and do not contradict each other since one model is developed in polyol solvents, capable of hydrogen bonding, while the other study is performed in non-hydrogen bonding solvents. 

Finally it is worth mentioning that a very detailed review has been published recently on the photophysics of Ru^II^-DPPZ compounds in diverse environments [101]. The reader should refer to that very good review for a more detailed discussion.

### 2.3. Ruthenium^II^ Complexes bearing DPPZ Analogues

**Figure 8:**
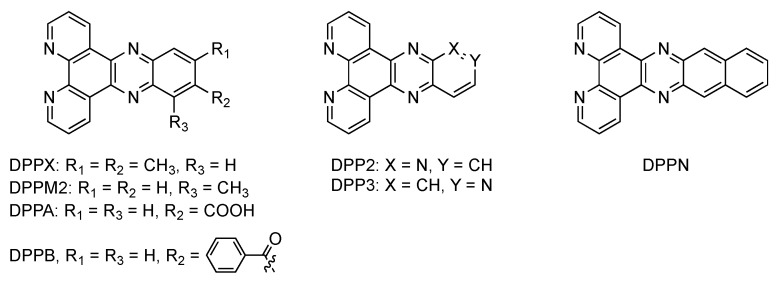
The seven DPPZ derivatives reported by Barton [11].

Since the discovery of this light-switch effect, and in order to understand the photophysical behavior of these ruthenium complexes, numerous analogues of DPPZ have been designed (Figure 8). 

In 1992, Barton published a paper with seven new DPPZ derivatives [11], but none of the resulting complexes could be considered as true “light switches”, even if some of them exhibited an emission increase by as much as 300 times upon binding to DNA. Some of these ligands will be discussed in the following sections of this review. 

In 2004, Lincoln [99] investigated two already reported complexes where the DPPZ moiety is substituted by a methyl group in position 10 (R_3_ = CH_3_), or by methyl groups in position 11 and 12 (R_1_ = R_2_ = CH_3_) [98,102,103]. The resulting complexes exhibit increased luminescence lifetimes as well as higher quantum yields in polyol solvents at room temperature compared to the parent compound [Ru(phen)_2_(DPPZ)]^2+^. Lincoln’s studies show that adding a methyl substituent decreases the hydrogen bonding of solvent molecules to the excited state. This decrease in hydrogen bonding is explained by means of entropy. The methyl substituents, in position 10, or 11 and 12, exhibit a steric hindrance that disarranges the whole “solvent cage” located around the molecule.

**Figure 9:**
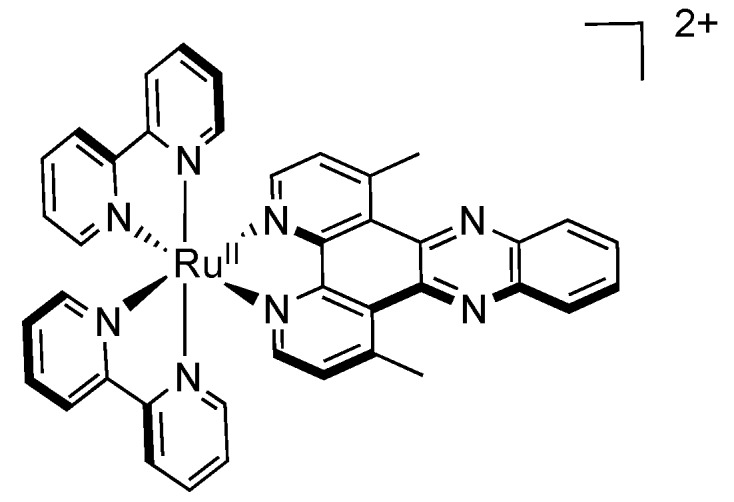
[Ru(bpy)_2_(dmDPPZ)]^2+^.

If these hydrophobic electron-donating methyl substituents are introduced on the 1,10-phenanthroline moiety of DPPZ (Figure 9), different properties can be observed [104].

Indeed by doing so, the optical properties as well as the redox properties can be strongly affected. Thus, these changes can affect the reactivity of the resulting complexes, in their ground state as well as in their excited state. Introducing methyl substituent allows to restore the luminescence of the corresponding ruthenium complex as compared to the parent compound based on the unsubstituted DPPZ. Moving towards electrochemistry, the introduction of donating groups shifts the reduction and oxidation potentials to more negative values, as already described for dimethyl substituted 2,2'-bipyridine and 1,10-phenanthroline [105,106]. 

Finally, more impressively, methyl substituents have a drastic influence on the excited state lifetime in methanol, where a typical increase of two or three times is generally observed, and an increase of 27 times is observed for [Ru(bpy)_2_(dmDPPZ)]^2+^ (dmDPPZ = 1,8-dimethyl-DPPZ), going from 30 ns for [Ru(bpy)_2_(DPPZ)]^2+^ to 820 ns for [Ru(bpy)_2_(dmDPPZ)]^2+^. This can be explained by a shielding effect from the methyl groups that prevent quenching by the solvent, which could be due to interactions with the phenazine core. 

**Figure 10:**
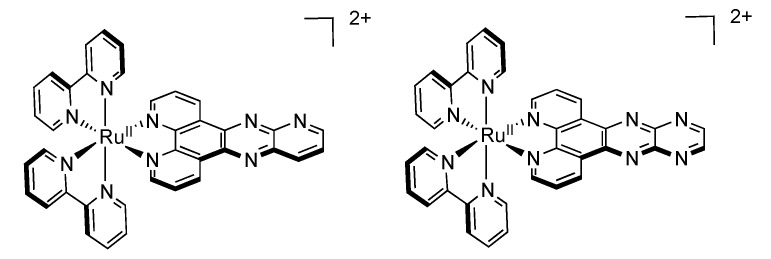
[Ru(bpy)_2_(dppp2)]^2+^ (left) and [Ru(bpy)_2_(dpqp)]^2+^ (right).

In 2010, Turro’s group reported the unusual photophysical properties of [Ru(bpy)_2_(dpqp)]^2+^ where dpqp stands for pyrazino[2',3':5,6]pyrazino[2,3-f][1,10]phenanthroline (Figure 10) [107]. Even if this complex is very much related to the parent compound [Ru(bpy)_2_(DPPZ)]^2+^, its photophysical behavior is dramatically different. Indeed, whereas [Ru(bpy)_2_(DPPZ)]^2+^ acts as a true light-switch, [Ru(bpy)_2_(dpqp)]^2+^ exhibits a strong luminescence in water at room temperature. Furthermore, the changes in the absorption spectra by varying the pH show that the complex [Ru(bpy)_2_(dpqp)]^2+^ can be protonated twice in its ground state whereas [Ru(bpy)_2_(DPPZ)]^2+^ can only be protonated once. For [Ru(bpy)_2_(DPPZ)]^2+^ protonation occurs on the phenazine ring, and the first protonation prevents a second protonation due to electronic repulsion. Analyzing the changes in luminescence intensity with pH allowed Turro’s group to extrapolate an apparent excited state pKa* of 2.1 for [Ru(bpy)_2_(dpqp)]^2+^, which is in the classical pKa range for related ruthenium complexes [108,109,110,111] (Table 1).

**Table 1:** Apparent excited state pKa for various complexes bearing bpy (2,2'-bipyridine), bpm (2,2'-bipyrimidine), bpz (2,2'-bipyrazine), TAP (1,4,5,8-tetraazaphenanthrene) and dpqp (pyrazino[2',3':5,6]pyrazino[2,3-f][1,10]phenanthroline) ligands [108,109,110,111].

Complex	pK_a_*(app)
[Ru(TAP)_3_]^2+^	3.5
[Ru(TAP)_2_(bpy)]^2+^	4
[Ru(TAP)(bpy)_2_]^2+^	3.1
[Ru(bpy)_2_(dpqp)]^2+^	2.1
[Ru(bpz)_3_]^2+^	3.30
[Ru(bpz)_2_(bpm)]^2+^	3.4
[Ru(bpm)_2_(bpz)]^2+^	3.5
[Ru(bpz)_2_(bpy)]^2+^	3.4
[Ru(bpy)(bpz)(bpm)]^2+^	3.1
[Ru(bpy)_2_(bpz)]^2+^	2.8
[Ru(bpm)_3_]^2+^	2.35
[Ru(bpm)_2_(bpy)]^2+^	2.25
[Ru(bpy)_2_(bpm)]^2+^	1.90

The luminescence that is observed probably arises from a ^3^MLCT excited state that is localized on the ancillary bpy ligand or on the portion of the dpqp ligand that is close to the ruthenium center. Indeed, it has been shown that the energy level of the MLCT involving the distal portion of the ligand is lower than the energy level corresponding to the orbital involving the phenazine core in [Ru(bpy)_2_(DPPZ)]^2+^. If emission arises from the distal portion of the dpqp ligand, it should be lower in energy than the emission of [Ru(bpy)_2_(DPPZ)]^2+^, which is not the case. Indeed, [Ru(bpy)_2_(dpqp)]^2+^ emission is closer to that of [Ru(bpy)_3_]^2+^. Furthermore, [Ru(bpy)_2_(dpqp)]^2+^ and [Ru(bpy)_3_]^2+^ present vibronic structure at 77 K as well as transient absorption feature that are similar.

**Figure 11:**
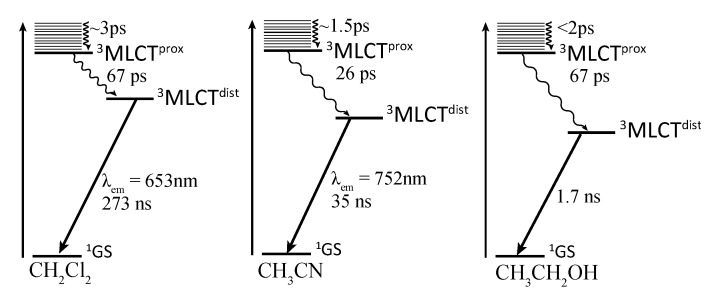
Jablonski diagrams of [Ru(bpy)_2_(dppp2)]^2+^ in CH_2_Cl_2_ (left), CH_3_CN (center), and CH_3_CH_2_OH (right). MLCT^dist^ stands for the distal portion of DPPZ while MLCT^prox^ stands for the proximal portion of DPPZ [112].

[Ru(bpy)_2_(dppp2)]^2+^ (Figure 10) [103,112,113] another [Ru(bpy)_2_(DPPZ)]^2+^ related complex, exhibits notable difference with the parent DPPZ compound. Indeed, [Ru(bpy)_2_(dppp2)]^2+^ cannot be considered as a light switch since it does not present a luminescence enhancement when bound to DNA, primarily due to the fact that the energy gap between the dark and bright states is important and precludes therefore the light-switch phenomenon. Even more interestingly, luminescence of complexes bearing dppp2 ligand exhibits a drastic dependence on the solvent nature. Indeed, [Ru(bpy)_2_(dppp2)]^2+^ luminesces at 752 nm in acetonitrile, while its emission is shifted to 653 nm in dichloromethane, and no luminescence is observed in ethanol (Figure 11).

Such an important effect is attributed to the presence of the nitrogen atom on the distal portion of the dppp2 ligand that is highly sensitive towards solvent polarity. When acetonitrile is used, the ^3^MLCT^dist^ state is highly stabilized, which results in a lower energy emission and a shorter lifetime. When a less polar solvent is used, *i.e.*, dichloromethane, the ^3^MLCT^dist^ state is less stabilized, and lies closer to the ^3^MLCT^prox^ state. The consequence of this weaker stabilization is an increased energy emission and a longer lifetime. 

**Figure 12:**
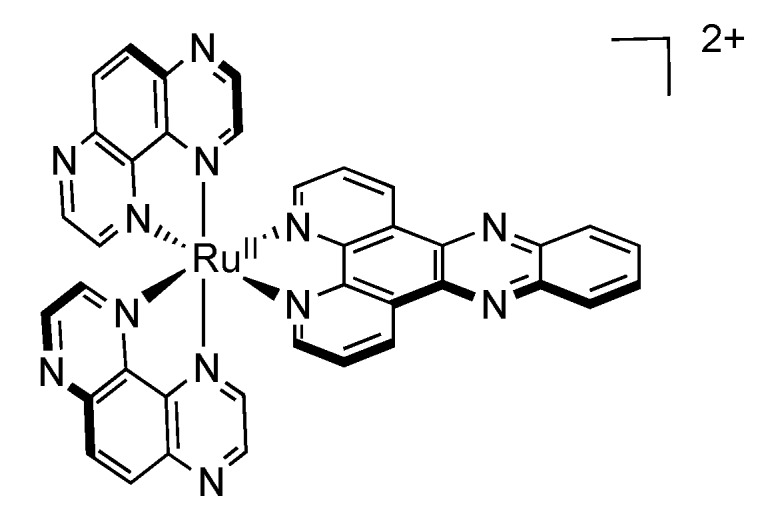
[Ru(TAP)_2_(DPPZ)]^2+^.

Finally, it is worth noting that if the 1,10-phenanthroline ligands in [Ru(phen)_2_(DPPZ)]^2+^ are substituted by more π-accepting ligands, such as 1,4,5,8-tetraazaphenanthrene (TAP), the resulting complex, *i.e.*, [Ru(TAP)_2_(DPPZ)]^2+^ (Figure 12) exhibits a luminescence in water as well as in organic solvents [34,114,115]. 

This behavior can be easily related to the fact that the first reduction wave occurs on the most π-accepting TAP ligand, meaning that the LUMO is localized on the TAP ligand and not on the DPPZ moiety anymore. Therefore, the lowest ^3^MLCT excited state corresponds to [Ru^III^(TAP)(TAP^•−^)(DPPZ)]^2+^*.

### 2.4. Ruthenium^II^ Complexes bearing Trischelating DPPZ Analogues

**Figure 13:**
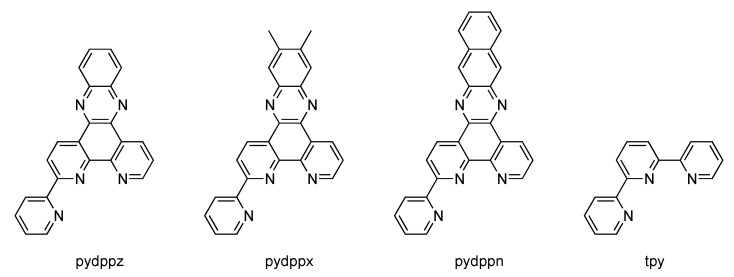
Tridentate ligands used in the studies performed by Turro.

In 2009, Turro’s group reported a very interesting ruthenium complex bearing a new tridentate ligand, pydppn (pydppn = 3-(pyrid-2'-yl)-4,5,9,16-tetraaza-dibenzo[a,c]naphthacene) (Figure 13) [116].

**Figure 14:**
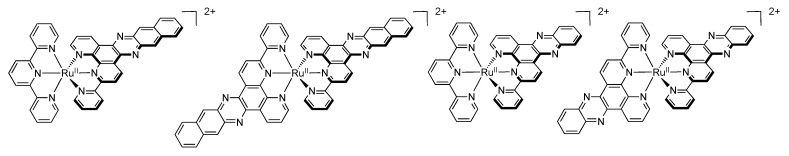
Ruthenium^II^ complexes bearing a pydppn ligand (left) or a pydppz ligand (right).

The resulting complexes [Ru(tpy)_n_(pydppn)_2-n_]^2+^ are able to generate ^1^O_2_ with almost 100% efficiency, but more interestingly, these complexes exhibit a photophysical scheme different from that of complexes bearing pydppz ligand (Figure 14). Whereas [Ru(tpy)_n_(pydppz)_2-n_]^2+^ complexes have a lowest lying ^3^MLCT excited state, with lifetimes ranging from 1 to 4 ns, complexes like [Ru(tpy)_n_(pydppn)_2-n_]^2+^ present a lowest lying ligand centered ^3^ππ* excited state that is localized on the pydppn ligand, with a lifetime of the order of 20 µs. 

**Figure 15:**
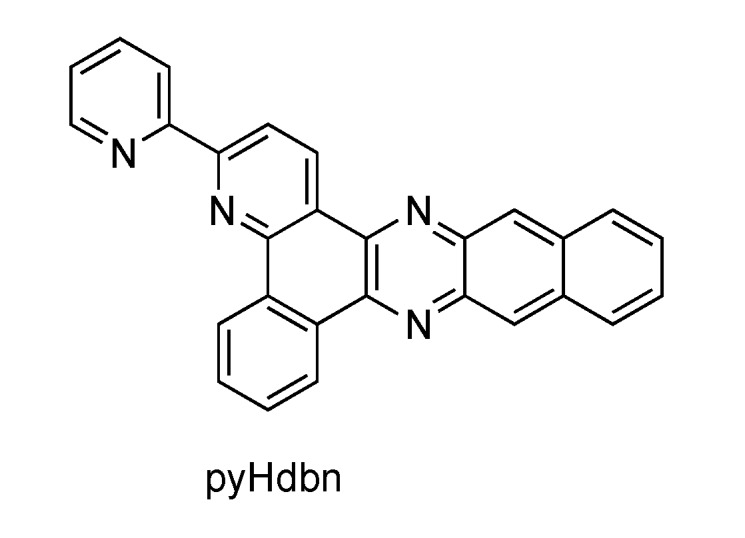
3-(Pyrid-2'-yl)-4,5,9,16-triazadibenzo[a,c]naphthacene (pyHdbn).

In parallel to these studies, Thomas’s group investigated the photophysical properties of [Ru(bpy)_2_(dppn)]^2+^, where dppn is benzo[i]dipyrido[3,2-a:2',3'-c]phenazine[117]. This study also revealed the existence of a lowest lying ligand centered ^3^ππ* excited state, localised on the dppn ligand. Also changing from pydppn to pydbn^−^ (where pyHdbn = 3-(pyrid-2'-yl)-4,5,9,16-triaza-dibenzo[a,c]naphthacene) (Figure 15) *i.e.*, a cyclometalated analogue of pydppn, the photophysical scheme is different [118]. Even if the extended π-systems pydppn and pydbn^−^ are very similar, the use of a negatively charged cyclometalating system increases the electron density on the ruthenium^II^ center, thus considerably changes the photophysical behavior of the corresponding complex.

**Figure 16:**
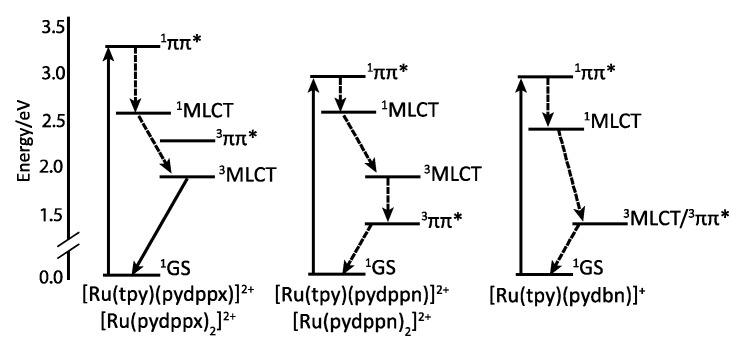
Jablonski diagrams for various ruthenium complexes [118].

This results in a lowest triplet excited state that presents a mixed character resulting from contribution of both ^3^MLCT and ^3^ππ* states (Figure 16), as shown by theoretical calculation. 

**Figure 17:**
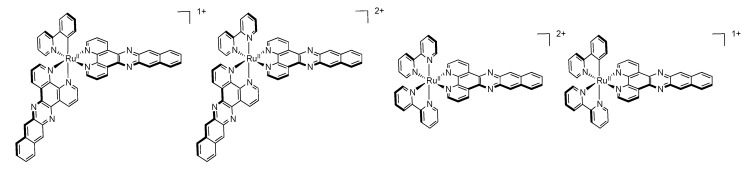
Ruthenium^II^ complexes bearing dppn, bpy and ppy ligands.

To further emphasize the influence of a cyclometalating unit, Turro’s group prepared a series of ruthenium^II^ complexes bearing dppn ligands and deprotonated 2-phenyl-pyridine ancillary ligand (ppy) [119] and compared these systems to their bpy analogues (Figure 17).

**Figure 18:**
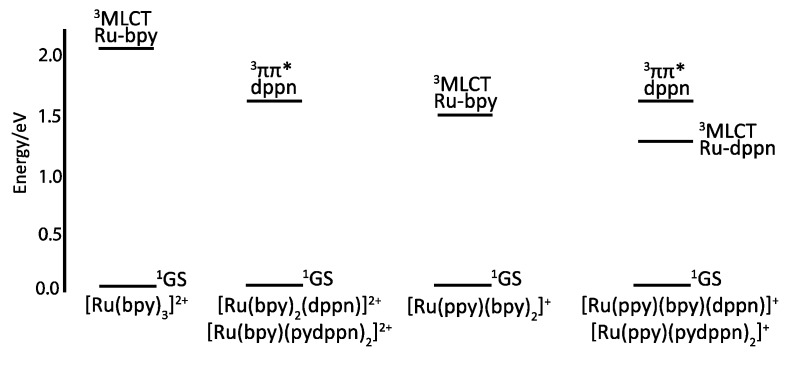
Relative energies of the low-lying triplet states of ruthenium^II^ complexes bearing dppn, bpy and ppy ligands [119].

It has been shown that even if [Ru(bpy)_2_(dppn)]^2+^ and [Ru(bpy)(dppn)_2_]^2+^ exhibit a long lived ^3^ππ* ligand centered excited state localized on the dppn ligand, the ppy analogues [Ru(ppy)(bpy)(dppn)]^+^ and [Ru(ppy)(dppn)_2_]^+^ exhibit a ^3^MLCT excited state, corresponding to a charge transfer from the ruthenium^II^ center to the dppn ligand (Figure 18), with a transient absorption lifetime of 25 and 45 ps respectively. 

**Figure 19:**
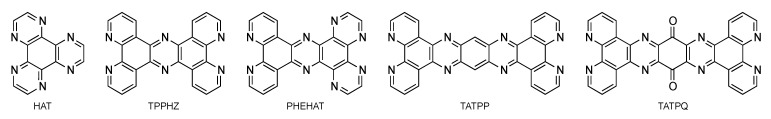
Examples of ligands with extended π-systems.

While DPPZ and its derivatives have drawn a lot of interest over the past years and will certainly continue to fascinate the scientific community in the future, new derivatives, more bulky or with the ability to bind multiple metal centers have been developed in order to extend the scope of application of ruthenium complexes. It is the case for example of ligands (Figure 19) such as 1,4,5,8,9,12-hexaaza-triphenylene (HAT) [120,121,122], tetrapyrido[3,2-a:2',3'-c:3'',2''-h:2''',3'''-j]phenazine (TPPHZ) [123,124], 1,10-phenanthrolino[5,6-b]1,4,5,8,9,12-hexaazatriphenylene (PHEHAT) [125], (9,11,20,22-tetraazatetrapyrido[3,2-a:2',3'-c:3'',2''-l:2''',3'''-n]pentacene (TATPP) [126,127,128,129], 9,11,20,22-tetra-azatetrapyrido[3,2-a:2',3'-c:3'',2''-l:2''',3'''-n]pentacene-10,21-quinone (TATPQ) [129]. 

These ligands, which will be discussed in the course of this review, all present characteristics that are required for optoelectronic applications, *i.e.*,: (i) a conjugated character that will allow efficient conjugation between metallic centers; (ii) a defined length, that is large enough to allow the connection of metal centers; (iii) a rigid and robust structure that offers a good stability to the system as well as avoids conformational problems. 

### 2.5. Mononuclear Ruthenium^II^-TPPHZ Complexes

Launay’s group first introduced TPPHZ in 1995 [123]. This ligand can be seen as a DPPZ moiety with two supplementary pyridine units. It offers the advantage of having two equivalent bidentate coordination sites, meaning that it can be used in the synthesis of binuclear compounds or for larger edifices. Due to its similarities with DPPZ, the mononuclear ruthenium complex [Ru(bpy)_2_(TPPHZ)]^2+^ exhibits the same light-switch properties as the DPPZ parent complex [123]. [Ru(bpy)_2_(TPPHZ)]^2+^ is not emissive in water, but when irradiated at 447 nm in dry acetonitrile, it exhibits intense luminescence at 616 nm with a lifetime of 628 ns under air, and 1,090 ns under inert atmosphere. As TPPHZ has an extended π-system, the resulting complex presents n-π* and π-π* transitions in the range 300–400 nm. Furthermore, TPPHZ having a better π-accepting character than 2,2'-bipyridine, the reduction process is facilitated (−0.87 V for [Ru(bpy)_2_(TPPHZ)]^2+^ compared to −1.31 V for [Ru(bpy)_3_]^2+^ *vs.* SCE) (Table 2) and is similar to the potential measured for [Ru(bpy)_2_(DPPZ)]^2+^] (E_red_ = −0.88 V *vs.* SCE). Over the years, TPPHZ has been involved in the synthesis of polynuclear complexes that are used in the fields of biology [12,130,131,132,133], polymer chemistry [133,134,135] dendrimers [136,137,138] and photo-catalysis [139,140]. 

Soon after the discovery of the light-switch effect of [Ru(bpy)_2_(TPPHZ)]^2+^, Launay’s group focused on the synthesis of mononuclear and binuclear TPPHZ Ru^II^ and Os^II^ complexes [127]. Binuclear complexes have been of considerable interest over the years as they allow energy and electron transfer between various metal centers. 

For example, in the heterodinuclear Ru-Os complex, a rapid (k > 10^9^ s^−1^) energy and/or electron transfer takes place from the Ru^II^ center to the Os^II^ center. This transfer, made possible by the TPPHZ bridging ligand, will be discussed in the next section of this review. 

**Table 2:** Redox potentials E(V) *vs.* SCE for various ruthenium^II^ complexes. Oxidation values are measured in acetonitrile and reduction potentials are measured in DMF *vs.* SCE unless otherwise noted. In parenthesis is the number of exchanged electrons.

Complex	Oxidation	Reduction
[Ru(bpy)_3_]^2+^ [91]	+1.27	−1.31 −1.50 −1.77
[Ru(phen)_3_]^2+^ [141]	+1.27	−1.35 −1.52
[Os(bpy)_3_]^2+^ [124,142]	+0.78	−1.30 −1.48 −1.78
[Ru(phen)_2_(TPPHZ)]^2+^ [137] ^a^	+1.34	−1.00 −1.38 −1.69
[Ru(bpy)_2_(TPPHZ)]^2+^ [124]	+1.33	−0.87 −1.33 −1.51 −1.73
[Os(bpy)_2_(TPPHZ)]^2+^ [124]	+0.88	−0.87 −1.24 −1.53 −1.79
[Ru(bpy)_2_(TPPHZ)Ru(bpy)_2_]^4+^ [124]	+1.34 (2)	−0.71 −1.31 (2) −1.51 (2) −1.72
[Os(bpy)_2_(TPPHZ)Os(bpy)_2_]^4+^ [124]	+0.89 (2)	−0.70 −1.21 (2) −1.44 (2)
[Ru(bpy)_2_(TPPHZ)Os(bpy)_2_]^4+^ [124]	+ 0.89 +1.33	−0.70 −1.27 (2) −1.48 (2) −1.66 −2.06

^a^ Oxidation and reduction potentials measured in acetonitrile.

It has been shown that the lowest ^3^MLCT excited state of [Ru(bpy)_2_(TPPHZ)]^2+^ is localized on the TPPHZ central unit. Moreover, the MLCT energy level depends strongly on the possible protonation or metalation of the terminal “phen” moiety.

**Figure 20:**
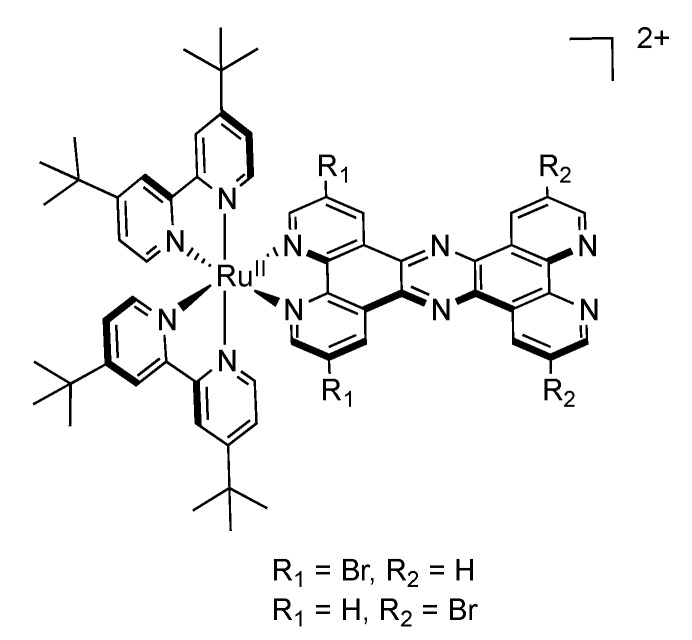
[Ru(tbbpy)_2_(TPPHZ)]^2+^ with bromine substituted TPPHZ.

In view of developing systems where the electron transfer can be directed, Rau’s group synthesized ruthenium complexes bearing a bromine substituted TPPHZ molecule [143]. Introduction of such accepting groups has no influence on the structural properties of the ligand itself, nor on the corresponding complex (Figure 20). 

Nonetheless, the coordination site is in that case important, depending if the bromine-substituted moiety is close to the ruthenium center or not. Bromine substituents far from the ruthenium center have little influence on the general behavior of the complex. On the contrary, the bromine groups close to the ruthenium ion exert an influence both on the redox potentials, and on the luminescence properties. Indeed, the emission maximum is red-shifted by 21 nm in dichloromethane and the lifetime of the brominated compound is increased by ca. 160 ns as compared to the unsubstituted complex (Table 3).

**Table 3:** Half-wave potentials E[V] *vs.* Fc/Fc^+^ in acetonitrile with 0.1M tetrabutylammonium tetrafluoroborate as supporting electrolyte [143].

Complex	λ_em_/nm (CH_2_Cl_2_)	λ_em_/nm (CH_3_CN)	(τ/ns) CH_2_Cl_2_	(τ/ns) CH_3_CN	Oxidation	Reduction
[Ru(tbbpy)_2_(TPPHZ)]^2+^ R_1_ = R_2_ = H	629	638	404.7	150.3	+0.83	−1.39 −1.88 −2.05 −2.26
[Ru(tbbpy)_2_(TPPHZ)]^2+^ R_1_ = Br, R_2_ = H	650	661	560.3	218.3	+0.89	−1.18 −1.87 −2.03 −2.18
[Ru(tbbpy)_2_(TPPHZ)]^2+^ R_1_ = H R_2_ = Br	633	645	390.0	92.1	+0.91	−1.25 −1.89 −2.06 −2.25

**Figure 21:**
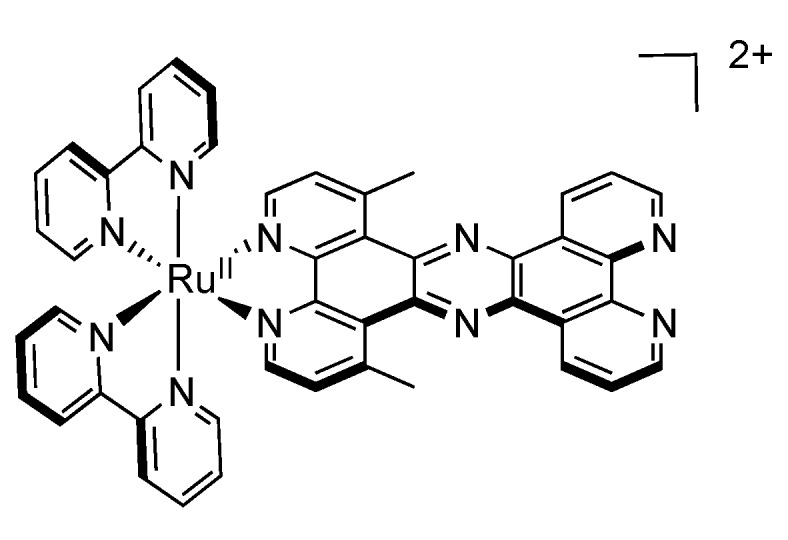
[Ru(bpy)_2_(dmTPPHZ)]^2+^.

Once again, as compared to the case of DPPZ bearing methyl substituents, when methyl substituents are introduced on the TPPHZ moiety, *i.e.*, when 4,7-dimethyl-TPPHZ (dmTPPHZ) is used, (Figure 21) the phenazine core is shielded by the methyl substituents, which makes solvent interaction less favorable. This shielding results in longer luminescence lifetimes, that are nonetheless less important than for the case of methyl substituted DPPZ.

### 2.6. Mononuclear Ruthenium^II^-TPAC Complexes

**Figure 22:**
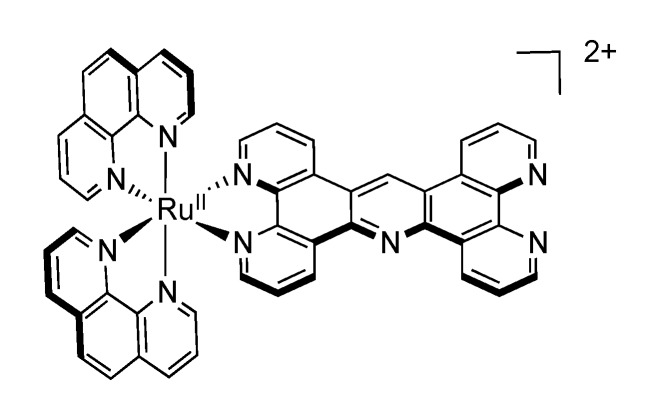
[Ru(phen)_2_(TPAC)]^2+^.

Seeing the drastic influence of the phenazine core in the DPPZ and TPPHZ ligands, Demeunynck *et al.* have focused their research on the development of the TPAC ligand (tetrapyrido[3,2-a:2',3'-c:3'',2''-h:2''',3'''-j]acridine) which is a TPPHZ analogue, where the central phenazine core is substituted by an acridine moiety [111,144,145] (Figure 22).

The resulting complex, [Ru(phen)_2_(TPAC)]^2+^ exhibits, as most of Ru^II^ polypyridyl complexes, intense absorption bands in the UV region and a relatively broad absorption band with a maximum at 448 nm (1.6 × 10^4^ M^−1^cm^−1^). More interestingly, after excitation at 450 nm, [Ru(phen)_2_(TPAC)]^2+^ exhibits an emission at 608 nm in acetonitrile and 613 nm in water. This constitutes a striking difference with the photophysics of the related TPPHZ or DPPZ complexes (those complexes do not emit in water) and emphasizes the fact that this central acridine or phenazine moiety plays a crucial role in the photophysical properties of the resulting complexes. Temperature dependent experiments highlighted that a slight variation of the emission maximum for [Ru(phen)_2_(TPAC)]^2+^ is observed when lowering temperature, going from 596 nm at 350K to 608 nm at 210K. The authors propose that two different MLCT excited states exist and are populated depending on the temperature [145]. They suggest that these two states correspond to a state where the electron is located on the phenanthroline moiety of the TPAC ligand at higher temperature and to a state where the electron is located more on the acridine moiety at low temperature. These two states could also explain the evolution of the luminescence lifetimes with temperature, meaning that two different luminescent states could participate to the deactivation process. It is worth noting that once again, when 1,10-phenanthroline ancillary ligands are substituted by π-accepting 1,4,5,8-tetraazaphenanthrene, the electron is located on the TAP ligand after photo-excitation, and not on the TPAC ligand anymore. 

In order to gain further clues on the processes that rule the photophysical scheme of [Ru(phen)_2_(TPAC)]^2+^, the effect of protonation on the spectroscopic properties of TPAC complexes bearing 1,10-phenanthroline or 1,4,5,8-tetraazaphenanthrene ancillary ligands has been examined. As no changes in the absorption spectra for the complexes [Ru(phen)_2_(TPAC)]^2+^ and [Ru(TAP)_2_(TPAC)]^2+^ are observed in the 1–14 pH range, it is necessary to go towards higher acidity and to use the Hammet acidity function H_0_ [146]. Changes in the photophysical properties are different if the ancillary ligand is a 1,10-phenanthroline or a 1,4,5,8-tetraazaphenanthrene. With [Ru(TAP)_2_(TPAC)]^2+^ the protonation of the ground state induces a drastic bathochromic shift in the absorption spectrum. After photo-excitation and relaxation, the electron is located on the TAP ligand. The energy level of this MLCT state is stabilized by the protonation of the nitrogen atom, which induces a bathochromic shift in the emission. A weak luminescence takes place at 900nm as compared to 642 nm for the unprotonated complex [111]. Regarding the complex bearing 1,10-phenanthroline ancillary ligands, a very small bathochromic shift is observed on the absorption spectrum for the protonated species. The main difference arises from the fact that the protonated [Ru(phen)_2_(TPAC)]^2+^ is not emissive. This could indicate that, for the unprotonated [Ru(phen)_2_(TPAC)]^2+^, photo-excitation promotes an electron from the Ru^II^ core towards the phenanthroline part of the TPAC ligand, giving rise to a luminophore that can be seen as Ru^II^-TPAC. On the other hand, the first protonation process takes place at the non-chelated phenanthroline moiety, causing therefore the electron to be localized either on the acridine moiety or on the non-chelated phenanthroline part, yielding a non luminescent ^3^MLCT state. 

### 2.7. Mononuclear Ruthenium^II^-PHEHAT Complexes

In order to explain the behavior of Ru^II^ complexes based on the extended PHEHAT ligand [125], it is worth reporting the photochemistry of complexes based on the HAT ligand (HAT = 1,4,5,8,9,12-hexaazatriphenylene) [147,148,149,150].

**Figure 23:**
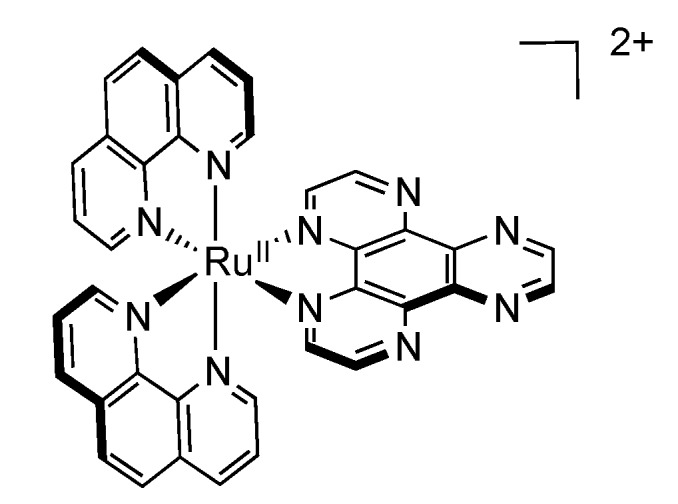
[Ru(phen)_2_(HAT)]^2+^.

As explained previously regarding the complex [Ru(TAP)_2_(DPPZ)]^2+^, the π-accepting properties of the TAP ligands have considerable influence on the photochemistry of the resulting complex. The HAT ligand, due to its supplementary fused pyrazine core, is even more π-accepting than TAP. The complex [Ru(phen)_2_(HAT)]^2+^ (Figure 23) for example has a ^3^MLCT excited state localized on the HAT moiety [150], due to its higher π-accepting character, leading to a bright state that can be attributed to [Ru^III^(phen)_2_(HAT^•−^)]^2+^*. 

**Figure 24:**
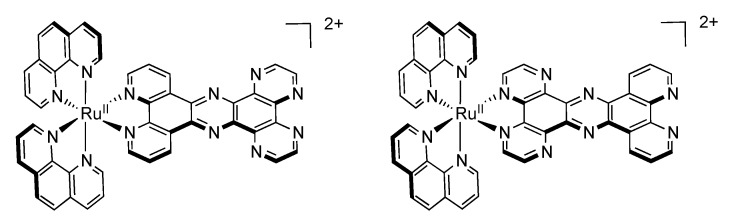
[Ru(phen)_2_(PHEHAT)]^2+^ (left) and [Ru(phen)_2_(HATPHE)]^2+^ (right).

The PHEHAT ligand itself exhibits the particularity of being symmetrical on an axis, and asymmetrical on the other. This dissymmetry is of tremendous interest for the photochemistry of the resulting complexes. Indeed, a different photochemical behavior is observed whether the ruthenium center is chelated via the “phen-like” moiety of the PHEHAT ligand, or whether it is chelated via the “HAT-like” moiety [151] (Figure 24).

When ruthenium is chelated via the “HAT-like” moiety, *i.e.*, [Ru(phen)_2_(HATPHE)]^2+^, the emission properties of the resulting complex are very similar to those of [Ru(phen)_2_(HAT)]^2+^, meaning that the ^3^MLCT excited state is localized on the HAT part of the PHEHAT ligand [151]. Thus, the “phen-like” moiety has little influence on the general photochemistry of the complex. More interestingly, when chelation is realized via the “phen-like” moiety, *i.e.*, [Ru(phen)_2_(PHEHAT)]^2+^, the photophysical scheme becomes more complicated and presents similarities with the scheme presented for [Ru(phen)_2_(DPPZ)]^2+^, as there are more than one excited state responsible for its photophysical behavior.

As it is the case for [Ru(bpy/phen)_2_(DPPZ)]^2+^, the excited state lifetime and the emission intensity of [Ru(phen)_2_(PHEHAT)]^2+^ in butyronitrile exhibit a maximum when the temperature decreases from 340 K to 220 K. It could thus be hypothesized that the photophysical scheme is the same as the one for [Ru(bpy/phen)_2_(DPPZ)]^2+^. Nonetheless, an important difference with DPPZ compounds is that the maximum emission wavelength changes with the temperature, while the literature concerning the photophysical scheme for Ru-DPPZ complexes does not report any change in the emission wavelength with temperature. This can be related to a photophysical scheme composed of two luminescent states [100]. 

The study of [Ru(phen)_2_(PHEHAT)]^2+^ at various temperatures shows indeed a change of the emission wavelength from 720 nm at 220 K to 630 nm at 340 K. These two wavelengths are attributed to two luminescent states, the first at 720 nm, of lower energy, and a second at 630 nm, higher in energy. 

**Figure 25:**
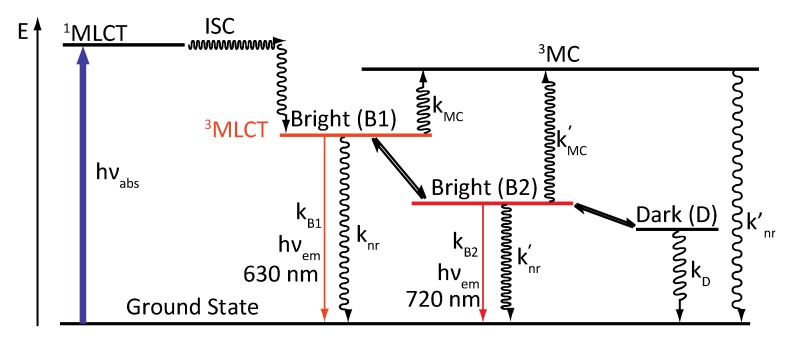
Photophysical scheme for [Ru(phen)_2_(PHEHAT)]^2+^.

Nevertheless, these two luminescent states cannot explain all the observed effects. In fact, when the lifetime is measured as a function of temperature, a lifetime of 150 ns at 220K is observed. The lifetime of the second luminescent state (lower in energy) is higher (281 ns). In regard to these observations, a third state, lower in energy and non luminescent, has to be introduced. This leads to a photophysical scheme (Figure 25) containing two luminophores in equilibrium with a dark state. Theoretical calculations are in agreement with the experimental data [152]. 

The two luminescent states are attributed to different parts of the ligand:

- B1, populated at high temperature should correspond to a ^3^MLCT state where the electron is located on the phen part of the ligand, whose characteristics are close to those of [Ru(phen)_3_]^2+^.
- B2, lower in energy, populated at low temperature, should correspond to a ^3^MLCT state where the electron is located on the “HAT” moiety of the ligand. This excited state presents similarities with the complex [Ru(phen)_2_(HAT)]^2+^.

As previously noted for DPPZ, changing the ancillary ligand from 1,10-phenanthroline to 1,4,5,8-tetraazaphenanthrene has also considerable influence on the photochemical behavior. For the complex [Ru(TAP)_2_(PHEHAT)]^2+^, the LUMO is once again localized on the TAP moiety, as indicated by cyclic voltammetry measurements, and not on the PHEHAT ligand anymore [145]. The complex [Ru(TAP)_2_(HATPHE)]^2+^ could only very recently be obtained and its photophysics has not been described up to now. The following table ends this part dedicated to mononuclear complexes by gathering their emission properties at room temperature (Table 4). 

**Table 4:** Emission properties of representative ruthenium^II^ mononuclear complexes.

Complex	λ_em_/nm CH_3_CN	τ/ns CH_3_CN deaerated	λ_em_/nm H_2_O	τ/ns H_2_O deaerated
[Ru(bpy)_3_]^2+^ [88]	620	855	626	630
[Ru(phen)_3_]^2+^ [153]	604	460	604	920
[Ru(bpy)_2_(DPPZ)]^2+^ [107]	631	750	---	---
[Ru(phen)_2_(DPPZ)]^2+^ [95]	607	663	---	---
[Ru(bpy)_2_(dpqp)]^2+^ [107]	618	921	617	582
[Ru(TAP)_2_(DPPZ)]^2+^ [115]	621		636	1090
[Ru(phen)_2_(TPPHZ)]^2+^ [137]	625	1250		
[Ru(phen)_2_(TPAC)]^2+^ [111]	608	253	613	839
[Ru(TAP)_2_(TPAC)]^2+^ [111]	624	1127	640	952
[Ru(phen)_2_(HAT)]^2+^ [151]	696	776	732	137
[Ru(phen)_2_(PHEHAT)]^2+^ [151]	662	262	---	---
[Ru(phen)_2_(HATPHE]^2+^ [151]	692	666	730	130

## 3. Binuclear Complexes

### 3.1. Binuclear TPPHZ Complexes

**Figure 26:**
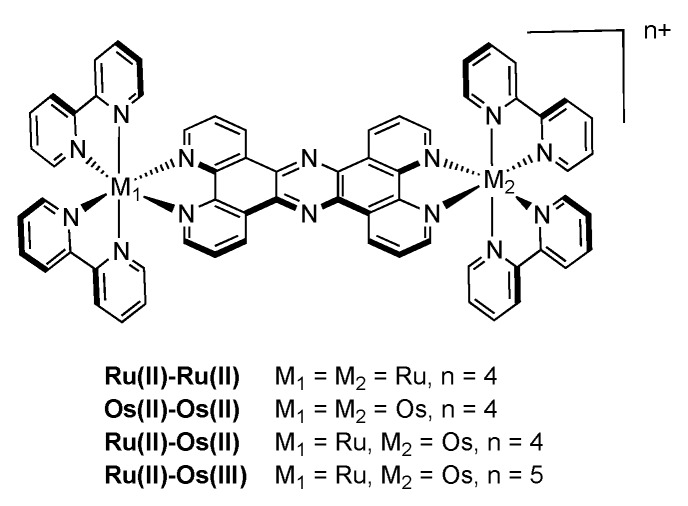
Binuclear TPPHZ complexes.

As one of the first bridging ligand, TPPHZ was rapidly exploited in the synthesis of binuclear [124,154,155] and polynuclear compounds [136,138,156]. Over the years, numerous studies about the photophysics of homodinuclear and heterodinuclear complexes based on ruthenium and osmium have been published (Figure 26). These studies include nanosecond, picosecond and femtosecond spectroscopies and the main results are depicted below.

#### 3.1.1. Ru^II^-Ru^II^ and Os^II^-Os^II^ Binuclear Complexes

**Figure 27:**
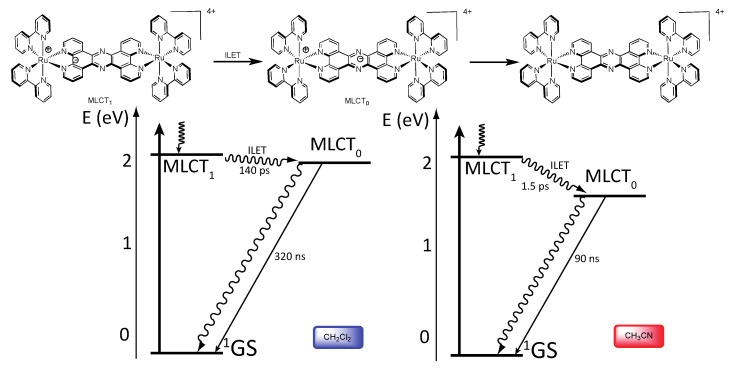
Photophysical mechanism for the Ru^II^-Ru^II^ TPPHZ binuclear complex in CH_2_Cl_2_ (**left**) and CH_3_CN (**right**) [155].

**Figure 28:**
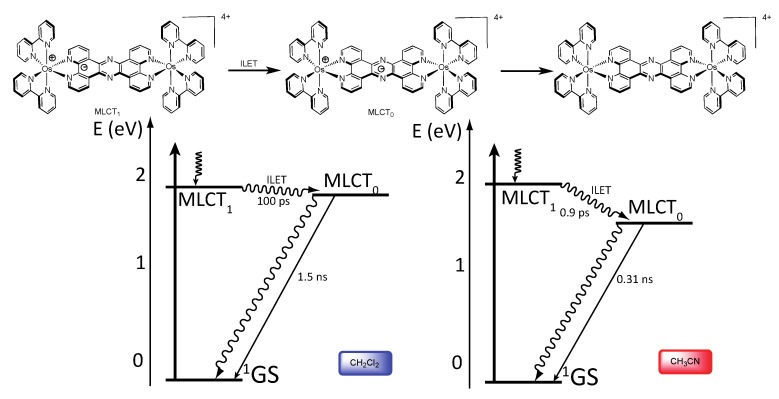
Photophysical mechanism for the Os^II^-Os^II^ TPPHZ binuclear complex in CH_2_Cl_2_ (**left**) and CH_3_CN (**right**) [155].

The photophysical data for the homodinuclear complexes [Ru(bpy)_2_(TPPHZ)Ru(bpy)_2_]^4+^ and [Os(bpy)_2_(TPPHZ)Os(bpy)_2_]^4+^, revealed the presence of two MLCT states, MLCT_1_ and MLCT_0_, both localized on the TPPHZ bridging ligand. MLCT_1_ is mostly localized on the 1,10-phenanthroline moiety of TPPHZ and is responsible for the absorption properties of the complexes, whereas MLCT_0_ is localized on the central pyrazine moiety. Emission arises from the MLCT_0_ state, lower in energy than MLCT_1_, stabilized in polar solvents. Experiments, carried out in dichloromethane and acetonitrile evidenced indeed the influence of polarity on the MLCT_0_ energy level. Femtosecond measurements show that after population of the MLCT_1_ state, an intraligand electron transfer (ILET) occurs, thus the electron located in the MLCT_1_ state is transferred towards the MLCT_0_, lower in energy. For the complex [Ru(bpy)_2_(TPPHZ)Ru(bpy)_2_]^4+^ this ILET occurs in 140 ps in dichloromethane and in 1.5 ps in acetonitrile. For the complex [Os(bpy)_2_(TPPHZ)Os(bpy)_2_]^4+^, this transfer occurs in 100 ps in dichloromethane and in 0.9 ps in acetonitrile. Both complexes then decay with lifetimes that can be obtained by nanosecond spectroscopy. As expected, the lifetime is much shorter for the Os^II^ complexes than for the Ru^II^ compounds, due to the heavy atom effect (Figure 27 and Figure 28).

#### 3.1.2. Ru^II^-Os^II^ Binuclear Complexes

The photophysical scheme for the heterobimetallic compound is more interesting as both metal centers can be considered as chromophores having each a MLCT_1_ and a MLCT_0_ state. The deactivation processes are therefore more complicated and multiple pathways for the energy transfer can be imagined. 

**Figure 29:**
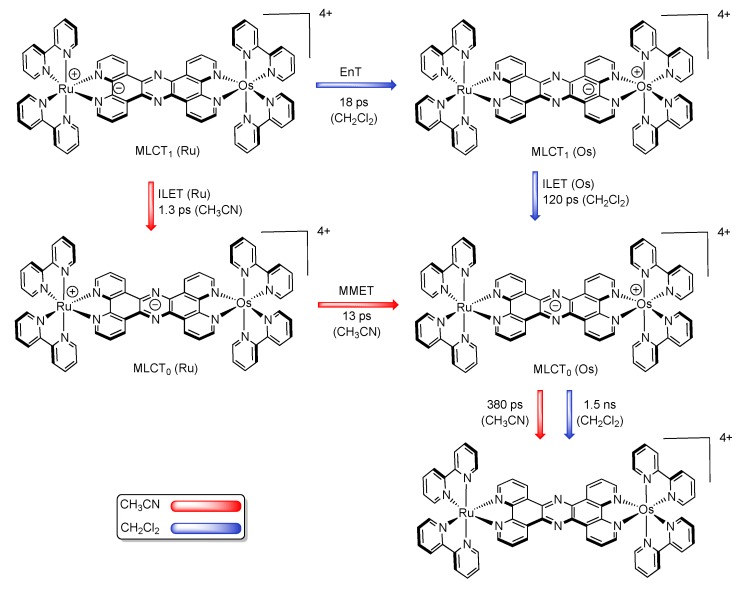
Schematic picture of the charge distribution along the photophysical pathway for the Ru^II^-Os^II^ TPPHZ binuclear complex in CH_2_Cl_2_ (blue) and CH_3_CN (red) [155].

Amazingly, Scandola *et al.* have found that the energy transfer process is different whether occuring in dichloromethane or in acetonitrile [155]. Indeed, in dichloromethane, a 18 ps energy transfer (EnT) from the Ru^II^ center towards the Os^II^ center at the MLCT_1_ level occurs. Then, an ILET process allows the relaxation from the Os^II^-based MLCT_1_ towards the MLCT_0_ in 120 ps. Finally, the excited MLCT_0_ state decays towards the ground state with a lifetime of 1.5 ns. In acetonitrile, the behavior is completely different, so that the first step is an ILET process that takes place in 1.3 ps and allows the relaxation of the Ru-based MLCT_1_ to the corresponding MLCT_0_ state. The second step is an energy transfer between the Ru^II^ center to the Os^II^ center at the MLCT_0_ level. This transfer can be seen as an excited-state metal-to-metal electron transfer (MMET) that occurs with a time constant of 13 ps. The last deactivation process corresponds to the relaxation to the ground state that occurs in 380 ps from the Os-based MLCT_0_ state (Figure 29).

#### 3.1.3. Ru^II^-Os^III^ Binuclear Complexes

**Figure 30:**
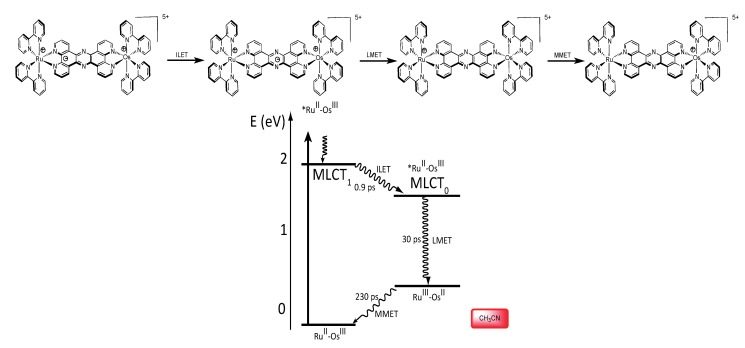
Photophysical mechanism for the Ru^II^-Os^III^ TPPHZ binuclear complex in CH_3_CN [155].

Finally, using the heterobimetallic complex [Ru^II^(bpy)_2_(TPPHZ)Os^III^(bpy)_2_]^5+^, obtained by oxidation of the osmium center (E = 0.89 V *vs.* SCE) in acetonitrile, three processes are observed. After excitation of the Ru^II^ chromophore, a two-step electron hopping process is observed, first by an ILET process that occurs in 2.1 ps from the MLCT_1_ state to the MLCT_0_ state, than by a ligand-to-metal electron transfer (LMET) that occurs in 30 ps. Finally, a MMET from the osmium center towards the ruthenium center occurs, and allows the population of the ground state with a time constant of 230 ps (Figure 30). 

### 3.2. Binuclear HAT Complexes

**Figure 31:**
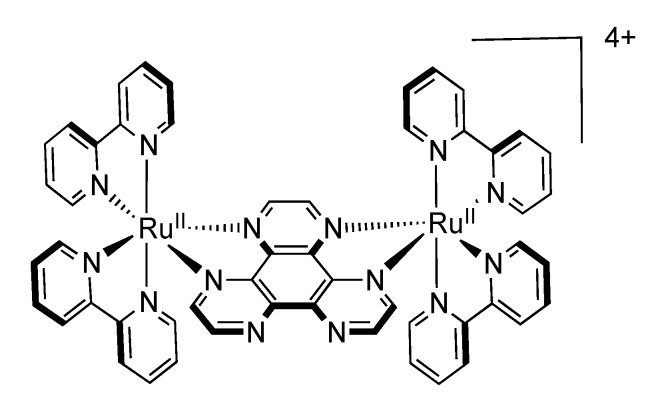
{[Ru(bpy)_2_]_2_(HAT)}^4+^.

Seeing how these metal centers are influenced by each others, it seemed interesting to discuss also complexes based on a smaller bridging ligand, *i.e.*, 1,4,5,8,9,12-hexaazatriphenylene [150]. It was possible, using HAT, to synthesize a wide variety of mono-nuclear and polynuclear complexes (Figure 31).

The photophysical properties of a series of selected mononuclear HAT complexes are gathered in Table 5. 

**Table 5:** Photophysical properties of a series of HAT complexes [150].

Complex	λ_em_/nm H_2_O	(τ/ns) H_2_O	λ_em_/nm CH_3_CN	(τ/ns) CH_3_CN
[Ru(bpy)_2_(HAT)]^2+^	742	104	703	620
[Ru(phen)_2_(HAT)]^2+^	732	137	698	817
[Ru(bpy)(TAP)(HAT)]^2+^	668	601	647	1764
[Ru(bpy)(HAT)_2_]^2+^	661	666	642	1810
Ru(TAP)_2_(HAT)]^2+^	608	315	595	102
[Ru(bpy)(HAT)_2_]^2+^	600	226	591	99
[Ru(HAT)_3_]^2+^	596	145	587	89

It is important to note that when a second ruthenium center is chelated on the central HAT ligand, the electronic communication is so important that the emission is shifted towards values superior to 800 nm for the bimetallic complexes and superior to 900 nm for the trimetallic compounds at room temperature.

Using electrochemistry (Table 6), it was possible to draw the following assessments. On one hand, the first oxidation always occurs on the Ru^II^ ion that is chelated to the better σ-donor ligand. On the other hand, the first reduction occurs on the better π-accepting ligand, and in this case, on the bridging HAT ligand in the monometallic complex, and also in the polymetallic complexes, since polycomplexation stabilizes the LUMO energy level localized on the HAT ligand. 

**Table 6:** Electrochemical data V *vs.* SCE for selected HAT complexes in acetonitrile with tetrabutylammonium hexafluorophosphate as supporting electrolyte [147,150].

Complex	Oxidation	Reduction
[Ru(bpy)_2_(HAT)]^2+^	+1.56	−0.84 −1.43 −1.63
[Ru(phen)_2_(HAT)]^2+^	+1.53	−0.86 −1.42 −1.69
{[Ru(bpy)_2_]_2_(HAT)}^4+^	+1.53 +1.78	−0.49 −1.06 ^a^
{[Ru(phen)_2_]_2_(HAT)}^4+^	+1.52 +1.78	−0.49 −1.07 ^a^
{[Ru(bpy)_2_]_3_(HAT)}^4+^	+1.61 +1.87 +2.12	−0.25 −0.58 −1.07
{[Ru(phen)_2_]_3_(HAT)}^4+^	+1.61 +1.88 +2.16	−0.30 −0.64 −1.12

^a^ Several other reduction waves are observed but are too close to be distinguished unambiguously.

In order to emphasize the influence of the second or third metal center, it is of interest to compare the first reduction of these complexes. Indeed, taking the complexes based on 2,2'-bipyridine and 1,4,5,8,9,12-hexaazatriphenylene ligands, the mononuclear [Ru(bpy)_2_(HAT)]^2+^ presents a first reduction wave at −0.84 V *vs.* SCE. Going from the mononuclear complex towards the binuclear complex {[Ru(bpy)_2_]_2_(HAT)}^4+^ and the trinuclear complex {[Ru(bpy)_2_]_3_(HAT)}^6+^ shifts the first reduction wave to −0.49 V and −0.25 V *vs.* SCE respectively. These important anodic shifts induced by the second and third metallic complexations indicate an important electronic communication in the HAT-based systems, with a ∆E_red_ of 0.35 V for the addition of a second Ru^II^ center, and a ∆E_red_ of 0.59 V when three Ru^II^ ions are present. 

### 3.3. Binuclear PHEHAT Complexes

**Figure 32:**
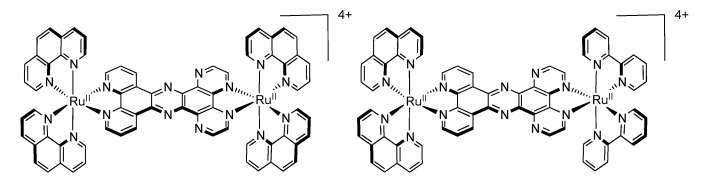
The complexes [Ru(phen)_2_(PHEHAT)Ru(phen)_2_]^4+^ (left) and [Ru(phen)_2_(PHEHAT)Ru(bpy)_2_]^4+^ (right).

We will first focus our discussion on the photophysical and electrochemical properties of two dinuclear complexes based on the non symmetrical PHEHAT ligand, [Ru(phen)_2_(PHEHAT)Ru(phen)_2_]^4+^ and [Ru(phen)_2_(PHEHAT)Ru(bpy)_2_]^4+^ (Figure 32) [157].

It has been shown, using electrochemistry measurements, that these two complexes present two one-electron oxidation at +1.34 V and +1.55 V *vs.* SCE (Table 7). Comparison with related ruthenium complexes, *i.e.*, [Ru(phen)_2_(PHEHAT)]^2+^ and [Ru(phen)_2_(HAT)]^2+^ allowed to assign the oxidation potential at +1.34 V to the oxidation of the Ru^II^ core chelated to the phenanthroline moiety of the PHEHAT ligand, and the potential at +1.55 V to the Ru^II^ ion chelated to the HAT moiety of the PHEHAT ligand. This indicates a stronger stabilization of the dπ orbital involving the Ru^II^ chelated on the HAT side than the one on the phenanthroline side. Furthermore, comparison with parent compounds indicates that the two ruthenium centers are poorly influenced by each other, suggesting a poor electronic interaction between these two centers. 

In reduction, two waves are observed at −0.68 and −1.06 (−1.07 for [Ru(phen)_2_(PHEHAT)Ru(bpy)_2_]^4+^) V *vs.* SCE that correspond to two successive additions of one electron on the PHEHAT ligand. Indeed, these potentials are not cathodic enough to be attributed to the reduction of the phenanthroline ligand since this reduction occurs at a reduction potential of −1.35 V *vs.* SCE. 

**Table 7:** Redox potentials E (V) *vs.* SCE for selected Ru^II^ complexes.

Complex	Oxidation	Reduction
[Ru(phen)_3_]^2+^ [17]	+1.27	−1.35 −1.52
[Ru(TAP)_3_]^2+^ [17]	+1.94	−0.75 −0.88 −1.10 −1.60 −1.80
[Ru(phen)_2_(HAT)]^2+^ [150]	+1.53	−0.86 −1.42 −1.69
[Ru(TAP)_2_(HAT)]^2+^ [150]	+2.02	−0.68 −0.86 −1.08
[Ru(phen)_2_(PHEHAT)]^2+^ [145]	+1.35	−1.00 ^a^ −1.25
[Ru(phen)_2_(HATPHE]^2+^ [145,151]	+1.56	−0.83 −1.01
[Ru(TAP)_2_(PHEHAT)]^2+^ [145]	+1.80	−0.75
[Ru(phen)_2_(PHEHAT)Ru(TAP)_2_]^4+^ [145]	+1.39 +2.10 ^a^	−0.52 −0.76
[Ru(phen)_2_(PHEHAT)Ru(phen)_2_]^4+^ [157]	+1.34 +1.55	−0.68 −1.06
[Ru(TAP)_2_(PHEHAT)Ru(phen)_2_]^4+^ [145]	+1.50 +1.86	−0.57 −0.79

^a^ poorly resolved.

**Figure 33:**
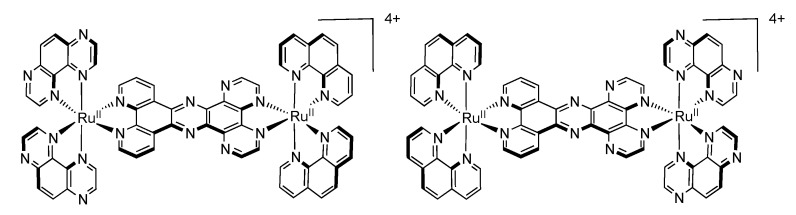
The complexes [Ru(TAP)_2_(PHEHAT)Ru(phen)_2_]^4+^ (left) and [Ru(phen)_2_(PHEHAT)Ru(TAP)_2_]^4+^ (right).

As shown earlier, it is possible in some dinuclear species to observe an electron or an energy transfer from one metallic center to the other. In such cases, after photo-excitation, relaxation to the lowest excited state occurs and, if the transfer is 100% efficient, emission is observed only from this lowest excited state [158,159]. The complex [Ru(phen)_2_(PHEHAT)Ru(phen)_2_]^4+^ exhibits an emission centered at 706 nm in acetonitrile. It is possible to consider this dinuclear complex as two separated entities, *i.e.*, [Ru(phen)_2_(PHEHAT)]^2+^ and [Ru(phen)_2_(HAT)]^2+^. In acetonitrile, these two species exhibit emission centered at 662 nm and 694 nm respectively. It is therefore proposed that the emission of [Ru(phen)_2_(PHEHAT)Ru(phen)_2_]^4+^ occurs from the Ru^II^ center chelated on the HAT side of the PHEHAT ligand. The same reasoning can be made for [Ru(phen)_2_(PHEHAT)Ru(bpy)_2_]^4+^ that emits at 714 nm in acetonitrile. This value can indeed be correlated to the emission of [Ru(bpy)_2_(HAT)]^2+^ that occurs at 703 nm in acetonitrile. These data indicate that an energy transfer takes place in these homonuclear complexes from the Ru^II^ center chelated on the phenanthroline part of the PHEHAT towards the Ru^II^ center chelated on the HAT side of the same ligand. Seeing the peculiar properties of this PHEHAT ligand, dinuclear species bearing 1,10-phenanthroline and 1,4,5,8-tetraazaphenanthrene ligands were studied, *i.e.*, [Ru(TAP)_2_(PHEHAT)Ru(phen)_2_]^4+^ and [Ru(phen)_2_(PHEHAT)Ru(TAP)_2_]^4+^ (Figure 33) [145].

For these complexes oxidation occurs at higher potentials than the ones described for the previously described dinuclear PHEHAT complexes, *i.e.*, [Ru(TAP)_2_(PHEHAT)Ru(phen)_2_]^4+^ exhibits two one-electron oxidation waves at +1.50 V and +1.86 V *vs.* SCE and [Ru(phen)_2_(PHEHAT)Ru(TAP)_2_]^4+^ exhibits two one-electron oxidation waves at +1.39 V and >2.10 V *vs.* SCE. Comparing these values with the mononuclear species allowed assigning the first wave to the oxidation of the Ru^II^ center bearing 1,10-phenanthroline ancillary ligands, and the second to the Ru^II^ center bearing 1,4,5,8-tetraazaphenanthrene ancillary ligands. Only the first and second reduction waves could be measured since at more cathodic potential, only irreversible or poorly resolved signals could be obtained. Two successive one-electron reduction waves at −0.55 V and −0.78 V *vs.* SCE have been assigned, as previously described, to two successive reductions of the PHEHAT ligand. Among these two complexes, only [Ru(TAP)_2_(PHEHAT)Ru(phen)_2_]^4+^ is luminescent at room temperature. The emission centered at 701 nm can be correlated to an emission arising from the Ru^II^ center chelated on the HAT part of the PHEHAT ligand. For [Ru(phen)_2_(PHEHAT)Ru(TAP)_2_]^4+^, no emission at room temperature can be observed, only luminescence at 604 nm in a rigid matrix at 77K is observed. This luminescence can be correlated to an emission arising from the [Ru(phen)_2_(PHEHAT)]^2+^ moiety. Indeed, at 77 K, the emission for [Ru(phen)_2_(PHEHAT)]^2+^ takes place at 598 nm, whereas the emission of [Ru(TAP)_2_(HAT)]^2+^ takes place at 575 nm at this temperature. Whereas the photophysical scheme for the previously described luminescent PHEHAT dinuclear complexes is quite straightforward, it is more challenging for the non emissive [Ru(phen)_2_(PHEHAT)Ru(TAP)_2_]^4+^. In the previous section of this review, we focused on the photophysical scheme of [Ru(phen)_2_(PHEHAT)]^2+^, presenting two bright states and one dark state, whose population varies strongly with temperature. In the case of [Ru(phen)_2_(PHEHAT)Ru(TAP)_2_]^4+^, since it is non luminescent at room temperature, one can postulate that the existence of such a dark state is also present. Furthermore, the use of TAP ligands tends to stabilize this excited state. To explain the non luminescence of [Ru(phen)_2_(PHEHAT)Ru(TAP)_2_]^4+^, it was suggested that at high temperature the excited state would deactivate extremely rapidly to a dark state and that at 77K in a solvent matrix, relaxation to lower states thanks to the solvent is disfavored, thus the bright state remains populated [160].

### 3.4. Binuclear TPAC Complexes

**Figure 34:**
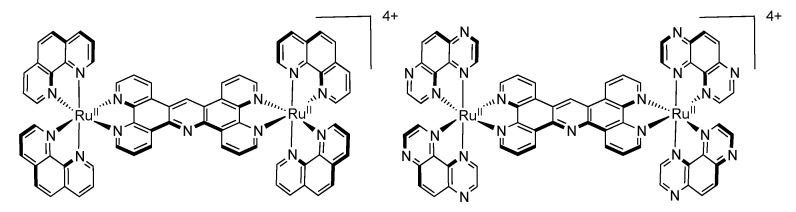
[Ru(phen)_2_(TAPC)Ru(phen)_2_]^4+^ (left) and [Ru(TAP)_2_(TPAC)Ru(TAP)_2_]^4+^ (right).

We will concentrate our discussion on two symmetrical luminescent binuclear TPAC complexes, *i.e.*, [Ru(phen)_2_(TPAC)Ru(phen)_2_]^4+^ and [Ru(TAP)_2_(TPAC)Ru(TAP)_2_]^4+^ (Figure 34) [111,144].

Comparing the oxidation potential of these binuclear complexes with that of the mononuclear analogues previously described shows that oxidation occurs at approximately the same potential. Indeed, [Ru(phen)_2_(TPAC)Ru(phen)_2_]^4+^ presents a bielectronic reversible oxidation at +1.31V *vs.* SCE (+1.33 V *vs.* SCE for its mononuclear analogue) while [Ru(TAP)_2_(TPAC)Ru(TAP)_2_]^4+^ presents a bielectronic reversible oxidation at +1.76 V *vs.* SCE (+1.70 V *vs.* SCE for its mononuclear analogue). This indicates that the addition of a second Ru^II^ ion on the TPAC ligand has only little influence on the oxidation values, suggesting therefore poor electronic communication between the two centers. Going towards reduction, the first reversible reduction wave for [Ru(phen)_2_(TPAC)Ru(phen)_2_]^4+^ occuring at −1.10 V *vs.* SCE can be attributed to the reduction of the TPAC ligand, while the second and third reduction waves at −1.32 V and −1.57 V *vs.* SCE are consistent with two simultaneous additions of two electrons to the 1,10-phenanthroline ligands. For [Ru(TAP)_2_(TPAC)Ru(TAP)_2_]^4+^, the first and second reduction waves that occur at −0.76 V and −0.92 V *vs.* SCE are assigned to two simultaneous additions of two electrons to the 1,4,5,8-tetraazaphenanthrene ligand, while the reduction of the TPAC ligand occurs at a potential of −1.26 V *vs.* SCE. Once again, emission of [Ru(TAP)_2_(TPAC)Ru(TAP)_2_]^4+^ involves a ^3^MLCT state localized on the π-accepting TAP ligand, which makes it hard to study the influence of the bridging TPAC ligand on the binuclear complex. For [Ru(phen)_2_(TPAC)Ru(phen)_2_]^4+^, the luminophore can be correlated to [Ru(phen)_2_(TPAC)]^2+^ or [Ru(phen)_3_]^2+^ due to the little influence exerted by the second Ru^II^ center. It should nonetheless be stressed out that the evolution of the emission lifetime with temperature is different for [Ru(phen)_2_(TPAC)Ru(phen)_2_]^4+^ than for the mononuclear [Ru(phen)_2_(TPAC)]^2+^. Moreover with the dinuclear compound no shift of the emission maximum with temperature is observed. Thus in that case it is proposed that [Ru(phen)_2_(TPAC)Ru(phen)_2_]^4+^ has only one emitting state whereas [Ru(phen)_2_(TPAC)]^2+^ has two. 

### 3.5. Binuclear TATPP Complexes

**Figure 35:**
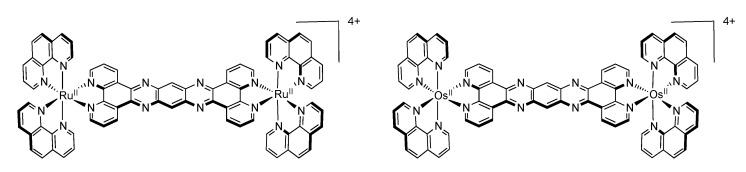
The two binuclear complexes, [Ru(phen)_2_(tatpp)Ru(phen)_2_]^4+^ and [Os(phen)_2_(tatpp)Os(phen)_2_]^4+^.

After numerous modifications that have been introduced over the years on the TPPHZ unit, the next step was to synthesize ligands with a still more extended π-system. Thus tatpp (tatpp = 9,11,20,22-tetraazatetrapyrido[3,2-a:2',3'-c:3'',2''-l:2''',3'''-n]pentacene) was developed for the synthesis of binuclear Ru^II^-Ru^II^ and Os^II^-Os^II^ complexes [161] (Figure 35). 

**Table 8:** Redox potentials V *vs.* SCE for selected Ru^II^ complexes with extended π-system measured in acetonitrile unless otherwise noted. In parenthesis is the number of exchanged electrons.

Complex	Oxidation	Reduction
[Ru(bpy)_2_(TPPHZ)Ru(bpy)_2_]^4+ a^ [124]	+1.34 (2)	−0.71−1.31 (2) −1.51 (2) −1.72
[Os(bpy)_2_(TPPHZ)Os(bpy)_2_]^4+ a^ [124]	+0.89 (2)	−0.70 −1.21 (2) −1.44 (2)
[Ru(bpy)_2_(TPPHZ)Os(bpy)_2_]^4+ a^ [124]	+ 0.89 +1.33	−1.27 (2) −1.48 (2) −1.66 −2.06
[Ru(phen)_2_(PHEHAT)Ru(bpy)_2_]^4+^ [157]	+1.34 +1.55	−0.68 −1.07
[Ru(TAP)_2_(PHEHAT)Ru(phen)_2_]^4+^ [145]	+1.50 +1.86	−0.57 −0.79
[Ru(phen)_2_(PHEHAT)Ru(TAP)_2_]^4+^ [145]	+1.39 +2.10	−0.52 −0.76
[Ru(phen)_2_(PHEHAT)Ru(phen)_2_]^4+^ [157]	+1.34 +1.55	−0.68 −1.06
[Ru(phen)_2_(TPAC)Ru(phen)_2_]^4+^ [145]	+1.31 (2)	−1.10 −1.32 (2) −1.57 (2)
[Ru(TAP)_2_(TPAC)Ru(TAP)_2_]^4+^ [145]	+1.76 (2)	−0.76 (2) −0.92 (2) −1.26 (1)
[Ru(phen)_2_(tatpp)Ru(phen)_2_]^4+^ [161]	+1.32 (2)	−0.26 −0.75 −1.32 (2)
[Os(phen)_2_(tatpp)Os(phen)_2_]^4+^ [161]	+0.85 (2)	−0.07 −0.24 −1.27 (2)

^a^ Reduction potentials measured in DMF.

Whatever the considered binuclear complex (Os-Os, Ru-Ru, TPPHZ, tatpp…) oxidation of the metal center appears as a single two-electron process, indicating therefore that the two metal centers are weakly coupled (Table 8). Oxidation for [Ru(phen)_2_(tatpp)Ru(phen)_2_]^4+^ and [Os(phen)_2_(tatpp)Os(phen)_2_]^4+^ occurs at +1.32 V and +0.85 V *vs.* SCE respectively. Looking into the reduction potentials indicates that the tatpp ligand is much easier to reduce than the TPPHZ analogue. Indeed, the first reduction is facilitated by 0.45 V for the ruthenium analogues, and by 0.63 V for the osmium analogues. Furthermore, using the tatpp ligand, two one-electron reduction processes could be observed on the bridging ligand instead of only one for the TPPHZ analogues. These two one-electron reduction processes are more positive for the osmium complex than for the ruthenium complex (0.19V for the first reduction and 0.51 V for the second). 

It is generally accepted [153] that for a Ru^II^ complex such as [Ru(bpy)_3_]^2+^, the energy of the MLCT transition can be directly correlated with the electrochemical potential obtained by cyclic voltammetry. This is not true anymore if the orbitals involved in the optical processes are not the same as those involved in the redox processes. The reduction at −0.26 V *vs.* SCE, anodically shifted by 0.52 V compared to the corresponding TPPHZ analogue, *i.e.*, [Ru(phen)_2_(TPPHZ)Ru(phen)_2_]^4+^ and 1.05 V compared to [Ru(bpy)_3_]^2+^ (Table 9), should thus have drastic influence on the MLCT absorption band if such a spectroelectrochemical correlation is present; the MLCT absorption band should indeed be red-shifted to roughly 700 nm, which is not the case as already observed for other extended systems [153]. 

DFT calculations showed that the lowest MLCT state involves an electron transfer from the metallic core towards a tatpp orbital that is mainly localized on the central position of the tatpp ligand and very little on the “phenanthroline-type” part of the tatpp ligand. This transition has however only a little oscillator strength, as compared to that of the MLCT state at higher energy. Indeed, the transition that is observed at higher energy corresponds to the promotion of one electron from the metallic core towards the “phenanthroline-type” part of the tatpp ligand, with a higher oscillator strength. This is in agreement with the MLCT absorption band localized experimentally around 450 nm, with a possible overlap of MLCT transition towards the ancillary phenanthroline ligand. These data confirm the fact that no spectroelectrochemical correlation can be established for such types of compounds. 

**Table 9:** Redox potentials V *vs.* SCE measured in acetonitrile for three Ru^II^ complexes with an increasing π-extended system.

Complex	Oxidation	Reduction
[Ru(bpy)_3_]^2+^ [91]	+1.28	−1.35 −1.55 −1.79
[Ru(phen)_2_(TPPHZ)Ru(phen)_2_]^4+^ [137]	+1.34 (2)	−0.78 −1.36 −1.52
[Ru(phen)_2_(tatpp)Ru(phen)_2_]^4+^ [161]	+1.32 (2)	−0.26 −0.75 −1.32 (2)

Looking at the photophysical behavior of the binuclear ruthenium^II^ and the binuclear osmium^II^ complexes is of interest since these complexes are non-luminescent. Transient absorption measurements allowed MacDonnell, Scandola, Campagna and coworkers to make the following assessments for the two binuclear tatpp complexes.

**Figure 36:**
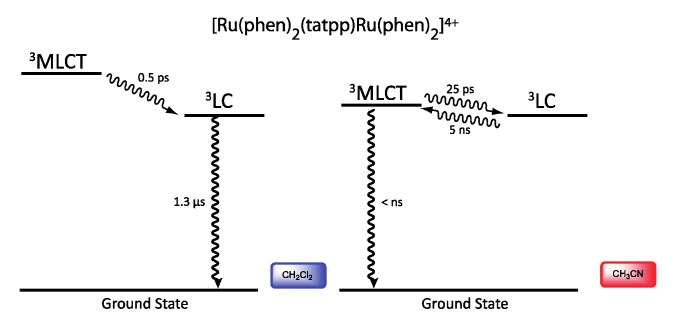
Energy level of the ^3^MLCT and ^3^LC states for the homodinuclear Ru^II^-tatpp complex [161].

Regarding the ruthenium^II^ complex, the transient formed at 580 nm decays in a few nanoseconds in deaerated acetonitrile and is long-lived (1.3 µs) in deaerated dichloromethane. By comparing the photophysics of the ruthenium complex with that of the tatpp ligand (as an adduct with an electronically innocent zinc ion), it was established that this transient at 580 nm corresponds to a tatpp LC triplet and not to an MLCT state [161]. Since this LC state is almost non polar, its relative energy is not expected to change by modifying the nature of the solvent, which corresponds to the observation. Therefore, the change in lifetime of the transient observed when going from dichloromethane to acetonitrile must result from the solvent-sensitive MLCT state which could play a role in the deactivation process of the LC state. In dichloromethane, the difference between the ^3^MLCT state and the ^3^LC state is so that the ^3^LC state is deactivated in an independent manner, with a lifetime of 1.3 µs. When acetonitrile is used, the ^3^MLCT state is stabilized, leading to a new deactivation pathway of ^3^LC via the ^3^MLCT in roughly 5 ns. The existence of such a low lying LC state, not common in the Ru^II^-polypyridyl complexes photochemistry, is related to the presence of the very much extended aromatic ligand (Figure 36).

**Figure 37:**
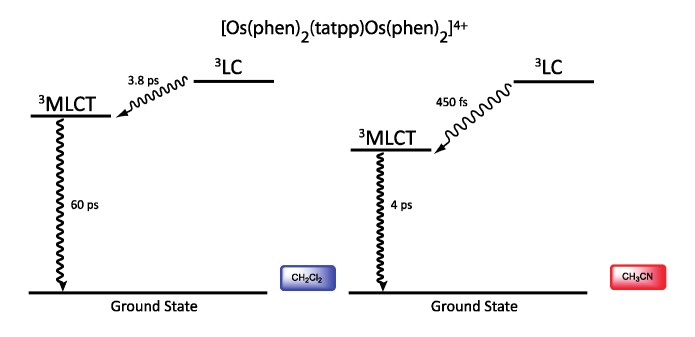
Energy level of the ^3^MLCT and ^3^LC states for the homodinuclear Os^II^-tatpp complex [161].

Regarding the osmium complex, the transient behavior is completely different. A decrease in optical density is observed between 500 and 560 nm that decays with a time constant of a few picosecond. Since osmium is used, it is accepted that the MLCT states are lower in energy than the corresponding MLCT for ruthenium analogues. Therefore, for the osmium complex, it seems plausible that the lowest lying excited state is a ^3^MLCT state that decays back to its ground state in 60 ps in dichloromethane and in 4 ps in acetonitrile. Transitions observed in transient absorption can therefore be assigned to a ^3^LC → ^3^MLCT conversion that occurs in 3.8 ps in dichloromethane and in 450 fs in acetonitrile (Figure 37). 

**Figure 38:**
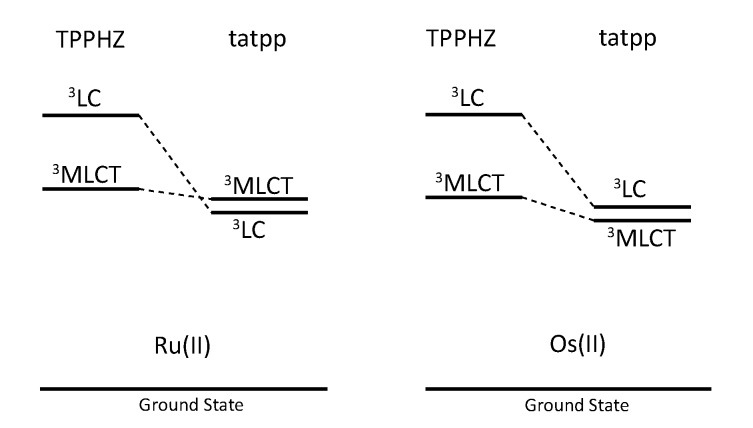
Relative energy of the ^3^MLCT and ^3^LC states of the Ru^II^ and Os^II^ complexes based on TPPHZ and tatpp [161].

Thus, using the same ligand, but with different metal centers, it was possible to assign two different excited states for the related complexes. When [Ru(phen)_2_(tatpp)Ru(phen)_2_]^4+^ is considered, the bridging ligand with an aromatic π-extended system decreases the MLCT energy level and more importantly the LC energy, leading to an inversion of the excited state as compared to TPPHZ related compounds, thus giving rise to a ^3^LC excited state (Figure 38). For [Os(phen)_2_(tatpp)Os(phen)_2_]^4+^, even if the excited state energies are lowered, no such inversion is observed, so that the excited state is still a ^3^MLCT one. 

Before discussing polynuclear species, it seemed important to note that throughout this study the communication between the two metallic centers is highly influenced by the bridging ligand. Comparing the first reduction potential for the binuclear complexes with that of the corresponding mononuclear complexes allows to draw the following conclusion (Table 10). For complexes bearing a HAT ligand, an anodic shift of 0.37 V could be observed, going from −0.86 V *vs.* SCE for [Ru(phen)_2_(HAT)]^2+^ to −0.49 V *vs.* SCE for [(phen)_2_Ru(HAT)Ru(phen)_2_]^4+^. On the other hand, an anodic shift of 0.22V could be observed when analyzing complexes based on TPPHZ. Indeed, the first reduction potential at −1.00 V *vs.* SCE for the mononuclear complex [Ru(phen)_2_(TPPHZ)]^2+^ was shifted to −0.78 V *vs.* SCE for the dinuclear species [(phen)_2_Ru(TPPHZ)Ru(phen)_2_]^4+^. The same comparison leads to a very small shift of only 0.04 V for the complexes based on tatpp. Taking these anodic shifts induced by the second metallic ion into consideration indicates that the electronic communication increases from tatpp (∆E_red_ = 0.04 V) to TPAC (∆E_red_ = 0.05 V) to PHEHAT (∆E_red_ = 0.16 V) to TPPHZ (∆E_red_ = 0.22 V) to HAT (∆E_red_ = 0.37 V).

**Table 10:** Redox potentials measured by cyclic voltammetry in acetonitrile *vs.* SCE for specific mononuclear complexes and their related binuclear complexes. In parenthesis is the number of exchanged electrons.

Complex	Oxidation, V *vs.* SCE	Reduction, V *vs.* SCE
[Ru(phen)_2_(HAT)]^2+^ [150]	+1.53	−0.86 −1.42 −1.69
[Ru(phen)_2_(TPPHZ)]^2+^ [137]	+1.34	−1.00 −1.38 −1.69
[Ru(phen)_2_(PHEHAT)]^2+^ [128]	+1.35	−0.84 −1.25
[Ru(phen)_2_(TPAC)]^2+^ [145]	+1.33	−1.15 −1.25 −1.35
[Ru(phen)_2_(tatpp)]^2+^ [127]	+1.33	−0.30 −0.83 −1.38
[(phen)_2_Ru(TPPHZ)Ru(phen)_2_]^4+^ [137]	+1.34 (2)	−0.78 −1.36(2) −1.52
[(phen)_2_Ru(HAT)Ru(phen)_2_]^4+^ [150]	+1.52 +1.78	−0.49 −1.07
[(phen)_2_Ru(TPAC)Ru(phen)_2_]^4+^ [145]	+1.31 (2)	−1.10 −1.32 (2) −1.57 (2)
[(phen)_2_Ru(PHEHAT)Ru(phen)_2_]^4+^ [157]	+1.34 +1.55	−0.68 −1.06
[(phen)_2_Ru(PHEHAT)Ru(bpy)_2_]^4+^ [157]	+1.34 +1.55	−0.68 −1.07
[Ru(phen)_2_(tatpp)Ru(phen)_2_]^4+^ [161]	+1.32 (2)	−0.26 −0.75 −1.32 (2)

## 4. Polynuclear Complexes

### 4.1. Polynuclear Complexes based on 2,3-dpp Ligand

**Figure 39:**
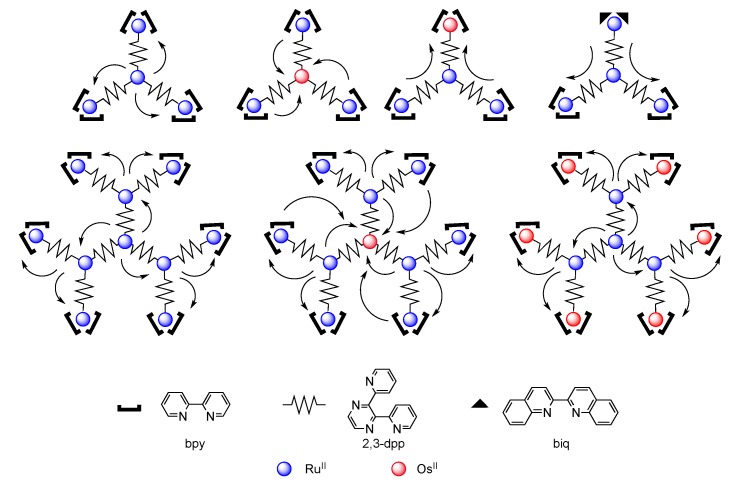
Schematic representation of the energy transfer in polynuclear complexes bearing 2,3-dpp bridging ligands [163,165].

Even if 2,3-dpp (2,3-bis-(2-pyridyl)pyrazine) (Figure 39) is not an aromatic ligand with an extended π-system, it seemed important to report the pioneer work that has been done with this ligand to understand the challenges behind the development of metallic dendrimers.

In a general way, dendrimers can be synthesized following two different approaches. The first one is called the convergent approach, or the “outside in” strategy and consists of connecting together preformed building blocks with a smaller one. The second strategy is referred to as divergent approach, or the “inside out” strategy, resulting in the reaction of a central core with different building blocks, creating therefore various layers, called generations. In order to link the different generations together, the different building blocks, in our case metallic ions or ligands, have to be able to make multiple bonds. Metallic cations such as ruthenium^II^ or osmium^II^ and bridging ligands such a 2,3-dpp were first used for this issue. Using these building blocks, numerous polynuclear species could be synthesized, whose properties depend drastically on the metallic ions and on the polypyridyl ligands that are used (Figure 39) [162,163,164,165,166].

**Figure 40:**
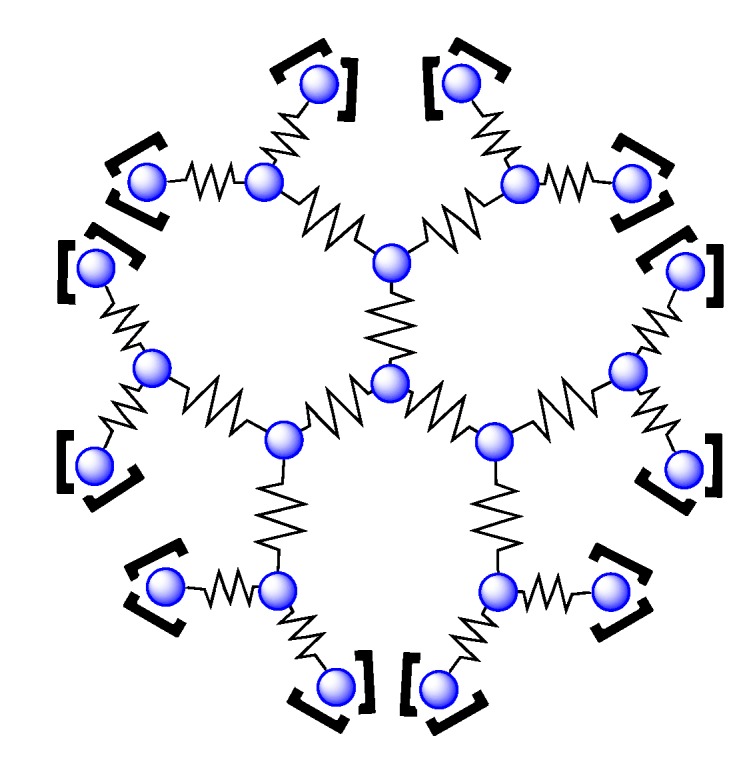
A Ruthenium dendrimer containing twenty-two metal centers [167].

Based on the 2,3-dpp ligand, Balzani, Campagna and Denti managed to synthesize and study the largest ruthenium dendrimer containing twenty-two metal centers [Ru{(µ-2,3-dpp)Ru[(µ-2,3-dpp)Ru{(µ-2,3-dpp)Ru(bpy)_2_}_2_]_2_}_3_]^44+^ (Figure 40) [167]. 

### 4.2. Polynuclear Complexes based on the HAT Ligand

1,4,5,8,9,12-hexaazatriphenylene also found rapid interest in the development of novel materials such as dendrimers due to its three coordination sites and to its stronger rigidity compared to 2,3-dpp, resulting from its 4 fused six-membered aromatic cycles.

**Figure 41:**
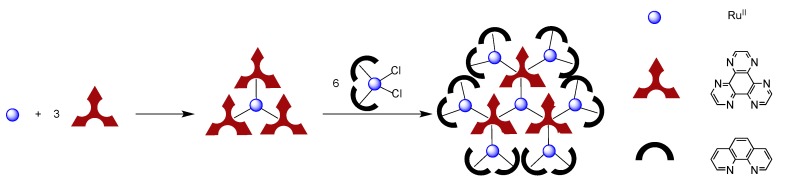
Pathway for the synthesis of the heptanuclear Ru^II^ core dendrimer [168].

**Figure 42:**
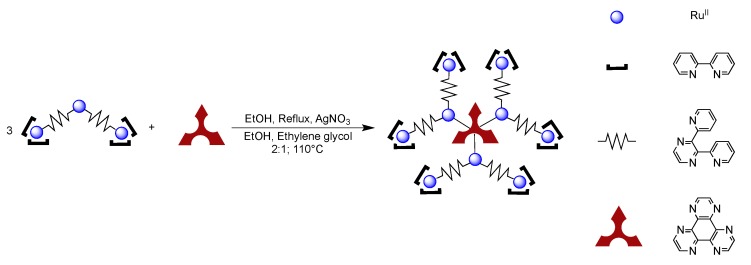
Pathway for the synthesis of the first ligand-cored nonanuclear dendrimer [171].

This ligand has allowed the synthesis of a heptanuclear Ru^II^ core dendrimer (Figure 41) [168,169], fully characterized by electrospray mass spectrometry and femtosecond transient absorption [170]. More interestingly, it has allowed the synthesis of the first ligand-cored nonanuclear dendrimer containing mixed polyazaaromatic bridging ligands (Figure 42) [171].

The heptanuclear complex can be synthesized in two steps. The first step is the reaction between the Wilkinson complex, [Ru(DMSO)_4_Cl_2_] and the HAT ligand, giving rise to the [Ru(HAT)_3_]^2+^ core which can be chelated to six other metallic ions. Thus, the second step consists in reacting [Ru(HAT)_3_]^2+^ with six equivalents of [Ru(phen)_2_Cl_2_], yielding the final {Ru(HAT)_3_[Ru(phen)_2_]_6_}^14+^. Although of great synthetic challenge, {Ru(HAT)_3_[Ru(phen)_2_]_6_}^14+^ didn’t find interest for applications in opto-electronics because of its non-luminescence. 

The synthesis of the nonanuclear dendrimer [HAT{Ru[2,3-dpp)Ru(bpy)_2_]_2_}_3_]^18+^, proceeds via the coordination of HAT with the previously synthesized [Cl_2_Ru{(2,3-dpp)Ru(bpy)_2_}_2_]^4+^ according to the convergent strategy. With its nine metal centers, this dendrimer exhibits two oxidation processes, one at +1.53 V *vs.* SCE and a second one at +2.13 V (Table 11). The first oxidation can be assigned to the simultaneous one-electron oxidation of the six peripheral ruthenium cations. The second oxidation process can be correlated to the oxidation of at least one ruthenium cation from the inner sphere. Since this oxidation potential is very close to the limit of the measurable oxidation window, it was not possible to confirm whether this process corresponds to a three electron process or a one electron process. Looking into the reduction potentials reveals a peculiar property of this nonanuclear ligand-core dendrimer. Indeed, two reversible processes, at −0.56 V and −0.68 V are observed. These two reduction processes are assigned to the reduction of the 2,3-dpp bridging ligands, and no reduction process is observed on the HAT core. It was proposed that this could be due to a shielding effect that prevents the contact between the HAT center and the electrode surface. This type of shielding was also observed for large dendrimers containing Co-phtalocyanines, Zn-porphyrins and iron-sulfur clusters as cores. [172,173,174]. 

**Table 11:** Redox potentials for polynuclear complexes in acetonitrile *vs.* SCE [171].

Complex	Oxidation, V *vs.* SCE	Reduction, V *vs.* SCE
[HAT{Ru[2,3-dpp)Ru(bpy)_2_]_2_}_3_]^18+^	+1.53(6) +2.13 ^a^	−0.56 −0.68 ^b^
[Cl_2_Ru{(2,3-dpp)Ru(bpy)_2_}_2_]^4+^	+0.82(1) +1.57(2)	−0.72(1) −0.88(1)
[HAT{Ru(bpy)_2_}_3_]^6+^	+1.61(1) +1.87(1) +2.12(1)	−0.25(1) −0.58(1)
[Ru{(2,3-dpp)Ru(bpy)_2_}_3_]^8+^	+1.53(3)	−0.56(1) −0.63(1)
[Ru{(2,3-dpp)Ru[(µ-2,3-dpp)Ru(bpy)_2_]_2_}_3_]^20+^	+1.53(6) +2.11 +2.44(3) ^c^	

^a^ The number of electrons has not been defined, ^b^ Both reductions involve approximately three electrons each, ^c^ Second and third processes are recorded in liquid SO_2_ [175].

### 4.3. Polynuclear Complexes based on the TPPHZ Ligand

TPPHZ, due to its two coordination sites has also rapidly found applications in the synthesis of larger structures such as polymers and dendrimers. Increasing the number of ruthenium centers also increased the number of stereoisomers that are present in the final polynuclear complex. Tremendous work has been performed by Campagna’s and MacDonnell’s group to isolate stereochemically pure dendrimers. Although very few data exists on the topic related to the photochemistry of stereochemically pure dendrimers (certainly because of the synthetic challenge), they showed that there are no significant differences in the photochemical behavior between various stereoisomers [137,138]. Indeed, for the complex {Ru[(TPPHZ)Ru(bpy)_2_]_3_}^8+^ it has been shown that an energy transfer occurs from the central Ru^II^ to the peripheral Ru^II^ atoms. It has also been demonstrated that the estimated distance of 22 Å between two terminal Ru^II^ atoms prevents any important coulombic interaction between these terminal Ru^II^ ions. Thus, the oxidation potential of the three peripheral ruthenium ions is achieved at the same potential. The same phenomenon is observed in reduction, as the first reduction wave corresponds to a three-electron process, meaning that the three TPPHZ molecules are reduced at the same potential. The same behavior is observed for all the studied stereoisomers of the tetranuclear compounds. 

### 4.4. Polynuclear Complexes based on the PHEHAT and TPAC Ligands

**Figure 43:**
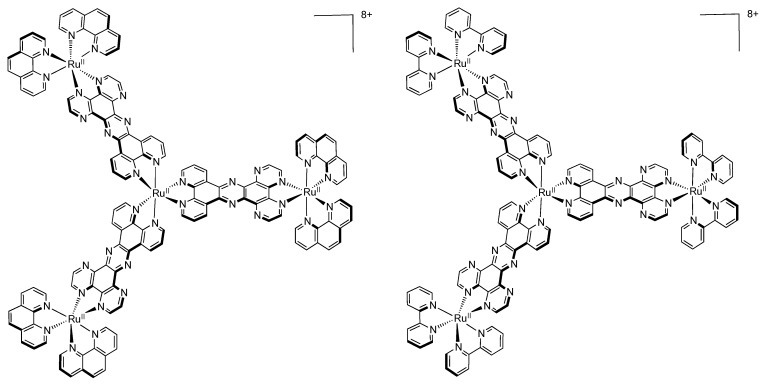
{Ru[PHEHAT-Ru(phen)_2_]_3_}^8+^ (left) and {Ru[PHEHAT-Ru(bpy)_2_]_3_}^8+^ (right).

As already mentioned, the PHEHAT ligand is a bridging ligand with an extended planar aromaticity that presents the particularity of being non-symmetrical. This non-symmetry is of tremendous interest since it could allow directing the energy transfer in the resulting multinuclear complexes. Up to now, only tetranuclear complexes, where the central ruthenium is coordinated to the “phen” moiety of the PHEHAT ligand could be synthesized [157]. The corresponding dendrimer having the central ruthenium ion coordinated to the HAT moiety of PHEHAT could still never be obtained. We will therefore focus our discussion on two tetranuclear complexes based on the PHEHAT ligand, namely {Ru[PHEHAT-Ru(phen)_2_]_3_}^8+^ and {Ru[PHEHAT-Ru(bpy)_2_]_3_}^8+^ (Figure 43). 

Electrochemistry measurements indicate a reversible one electron oxidation wave at +1.38 V *vs.* SCE and +1.34 V *vs.* SCE for {Ru[PHEHAT-Ru(phen)_2_]_3_}^8+^ and {Ru[PHEHAT-Ru(bpy)_2_]_3_}^8+^ respectively, followed by a reversible three electron oxidation wave at +1.56 V *vs.* SCE and +1.54 V *vs.* SCE. Taking into account the number of electrons involved in each oxidation wave, and comparing the oxidation potentials with that for the corresponding mononuclear complexes, allows attributing the first oxidation wave to the abstraction of one electron from the Ru^II^ core and the second oxidation to the abstraction of one electron from each Ru^II^ ion present in the peripheral region. 

**Figure 44:**
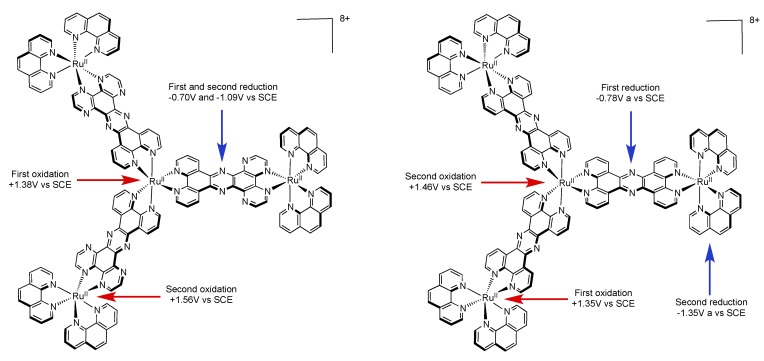
Schematic representation of the oxidation and reduction processes in PHEHAT and TPPHZ tetranuclear complexes.

On the other hand, two distinct three electrons reduction waves are observed in the region comprised between 0 and −1.20 V *vs.* SCE. These waves are attributed to the successive addition of two electrons to one PHEHAT ligand since the two reduction potentials are not cathodic enough to reduce a 1,10-phenanthroline ligand (Figure 44). A different scenario was observed for TPPHZ based complexes for which the second electron is added on the 1,10-phenanthroline ligand [124,136]. 

Going more deeply into electrochemistry, an anodic shift can be observed from mononuclear PHEHAT complexes to polynuclear complexes, from −0.84 V *vs.* SCE for [Ru(phen)_2_(PHEHAT)]^2+^ to −0.69 V *vs.* SCE for the polynuclear complex, in agreement with the data for dinuclear analogue (Table 12). 

**Table 12:** Redox potentials measured by cyclic voltammetry in acetonitrile *vs.* SCE at room temperature, with 0.1M ^t^Bu_4_N^+^ClO_4_^−^ as supporting electrolyte and a Pt working electrode. In parenthesis is the number of exchanged electrons.

Complex	Oxidation, V *vs.* SCE	Reduction, V *vs.* SCE
[Ru(phen)_3_]^2+^ [141]	+1.27(1)	−1.35(1) −1.52(1)
[Ru(phen)_2_(TPPHZ)]^2+^ [137]	+1.34(1)	−1.00(1) −1.38(1) −1.69(1)
[Ru(phen)_2_(PHEHAT)]^2+^ [145]	+1.35	−0.84 −1.25
[(phen)_2_Ru(TPPHZ)Ru(phen)_2_]^4+^ [137]	+1.34(2)	−0.78(1) −1.36(2) −1.52
[(phen)_2_Ru(PHEHAT)Ru(phen)_2_]^4+^ [157]	+1.34(1) +1.55(1)	−0.68(1) −1.06(1)
[(phen)_2_Ru(TPPHZ)Ru(bpy)_2_]^4+^ [137]	+1.34(1) +1.55(1)	−0.68(1) −1.07(1)
{Ru[(TPPHZ)Ru(phen)_2_]_3_}^8+^ [137]	+1.35(3) +1.46(1)	−0.78(3) −1.35 (3) −1.54
{Ru[(PHEHAT)Ru(phen)_2_]_3_}^8+^ [157]	+1.38(1) +1.56(3)	−0.70(3) −1.09(3)
{Ru[(PHEHAT)Ru(bpy)_2_]_3_}^8+^ [157]	+1.34(1) +1.54(3)	−0.69(3) −1.07(3)

**Figure 45:**
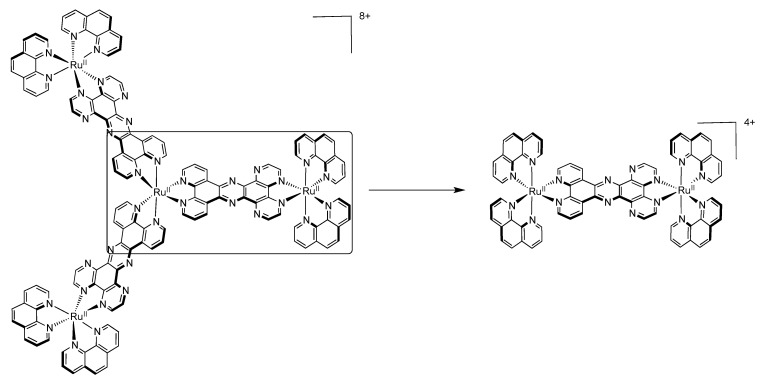
Schematic representation of a dinuclear subunit present in the dendritic compound.

These tetranuclear compounds can be seen as three identical dinuclear complexes (Figure 45) and it is thus interesting to compare their luminescent properties in acetonitrile. 

{Ru[PHEHAT-Ru(phen)_2_]_3_}^8+^ exhibits an emission maximum at 708 nm whereas {Ru[PHEHAT-Ru(bpy)_2_]_3_}^8+^ exhibits an emission maximum at 716 nm. To assess whether or not an energy transfer is present in these polynuclear species, it is necessary to compare their emission maximum with the one for the corresponding mononuclear species. Indeed, [Ru(phen)_2_(PHEHAT)]^2+^ has a maximum centered at 662 nm whereas [Ru(phen)_2_(HAT)]^2+^ has a maximum centered at 694 nm and [Ru(bpy)_2_(HAT)]^2+^ at 703 nm. Based on these emission maxima, it clearly appears that the emission must arise from the Ru-HAT moiety, thus involving the Ru^II^ center on the HAT part of the PHEHAT bridging ligand. This is clear-cut evidence that an energy transfer process takes place from the inner to the outer metallic units of the tetranuclear compounds. 

Up to now, we have focused this review on the photophysics and electrochemistry of various ruthenium^II^ complexes based on ligands or bridging ligands with extended π-systems. We have shown that an electron transfer could occur and could be directed by means of the metal center or by using π-accepting bridging ligands. We will now discuss how to take advantage of these properties to design systems with applications in photo-catalysis, focusing principally on hydrogen production. 

## 5. Water Splitting and Hydrogen Production

Before focusing our discussion on binuclear complexes based on polyazaaromatic extended ligands as intramolecular catalysts for the production of hydrogen or for water splitting, it seemed important to emphasize the fact that numerous systems capable of generating hydrogen were described over the time. In fact, since the discovery of [Ru(bpy)_3_]^2+^ as a possible photo-sensitizer for the splitting of water [176,177], numerous researches have led to the development of systems capable of producing hydrogen. In 1977, Lehn and Sauvage [178] introduced a four-component system, based on a ruthenium polypyridine sensitizer linked to a rhodium bipyridine complex that acts as an electron relay, an electron donor such as triethanolamine and colloidal platinum that acts as the hydrogen generating catalyst. This type of system, although still used, depends strongly on diffusion controlled processes. During the 80’s, Meyer [179,180] and Grätzel [181] focused on the “blue-dimer”, *i.e.*, a ruthenium oxo-bridged dinuclear complex that efficiently catalyzes water oxidation. In the 90’s, Brewer and coworkers designed a system based on ruthenium^II^, iridium^III^ or rhodium^III^ that could act as molecular devices for photoinitiated electron collection, capable of storing photo-electrons on their bridging ligand [182,183]. Later on Nocera *et al.* introduced a molecular photocatalyst capable of generating hydrogen from hydrohalic acid solutions [184]. This molecular photocatalyst consists of a two-electron mixed-valence dirhodium compound that leads, after absorption of light and breaking of two Rh^II^-X bonds, to the active rhodium catalyst which finally reacts with hydrohalic acids. Nocera *et al.* then developed oxygen evolving complexes in the same water splitting context, based on cobalt and phosphate [185], or nickel and borate [186,187] that self-assemble *in situ* to form the desired catalyst [188]. Since then, numerous publications have focused on the use of molecular complexes for water-splitting catalysis [189,190,191,192,193,194]. Meyer, Sun and others developed mononuclear ruthenium catalysts [195,196,197], as well as ruthenium dimers [198,199,200,201] for catalytic water oxidation. Sakai focused on platinum^II^ complexes, or hybrid ruthenium^II^ – platinum^II^ systems for hydrogen production from water [202,203]. Sun *et al.* ended up with a mononuclear ruthenium complex that has a turnover frequency superior to 300 s^−1^, very close to that of the oxygen-evolving complex found in photosystem II [204]. Very recently, Collomb *et al.* have reported the use of a polypyridyl photocatalyst based on two ruthenium^II^ chromophores and one rhodium^III^ catalytic center that produces hydrogen in aqueous solution [205]. 

### 5.1. Complexes based on Ligands with Extended Aromaticity as Catalysts for Hydrogen Production

In this section, we will focus our discussion on intramolecular photocatalysts that are used for hydrogen production. These photocatalysts are constituted of a photo-active center, generally a Ru^II^ dye, capable of absorbing visible light, a molecular bridge that acts as an electron relay or reservoir and a catalytically active center, *i.e.*, a redox active metal center. 

One of the first intramolecular catalyst reported in the literature has been designed using TPPHZ ligand [139]. The general mechanism of activity for this photocatalyst can be described as follow: first, the ruthenium dye absorbs a photon in the visible region, allowing the complex to reach the ^1^MLCT excited state. After intersystem crossing to the ^3^MLCT excited state, the electron is transferred to the bridging ligand, then towards the palladium center allowing a first reduction to occur. Triethylamine, present in the system, is added as a sacrificial reductant in order to facilitate the reduction of the formally generated Ru^III^ into Ru^II^. This reduction regenerates the initial system and the mechanism can be repeated until a Pd^0^ center is obtained. The reduction of the palladium center is generally accompanied by ligand substitution. 

**Figure 46:**
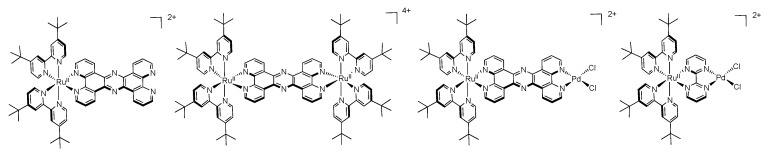
Complexes used by Vos [139].

In their seminal 2006 paper [139], Vos et al. investigated the different parameters that have to be taken into account in order to produce hydrogen. First, using the mononuclear, palladium free, TPPHZ complex, they observed only low hydrogen production, meaning that the mononuclear ruthenium complex is a poor photocatalyst. Second, using the homodinuclear complex, only very small amounts of hydrogen could be produced, meaning that the presence of a redox active center is required for photocatalytic hydrogen production. Typical turnover numbers TON for the mononuclear or homodinuclear complex were of only 0.56. Replacing the bridging ligand TPPHZ for the smallest polypyridine-type bridging ligand, i.e., bipyrimidine, also led to a loss of catalytic activity. Finally, it has been shown that concentration of triethylamine has also a strong influence on the amount of hydrogen that is photocatalytically produced. On a general perspective, it has been demonstrated that the presence of all three components, i.e., a Ru^II^ photoactive center, a bridging ligand with extended aromaticity and a catalytic redox center, is required for the design of an effective photocatalyst for hydrogen production. Using their system based on [Ru(tbbpy)_2_(TPPHZ)PdCl_2_]^2+^ (tbbpy = 4,4'-di-tert-butyl-2,2'-bipyridine) (Figure 46), they managed to obtain a turnover number of 56.4 mol of hydrogen per mol of catalyst, with a turnover frequency of circa 1,700 nmol.min^−1^. 

The researches then developed in view of increasing the efficiency of the system and understanding the mechanism of electron transfer and relay, in order to find and design better photocatalysts. 

**Figure 47:**
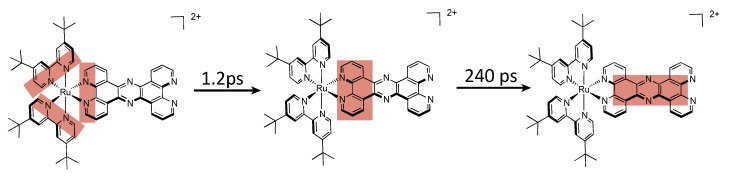
Charge distribution along the photophysical pathway for [Ru(tbbpy)_2_(TPPHZ)]^2+^ [206].

In that context, more fundamental studies allowed a deeper insight in the behavior of these systems [140,206]. Resonance Raman indicates that the initial excitation is delocalized over the tbbpy and TPPHZ ligand and is followed by an inter-ligand hopping mechanism that transfers the electron on the phenanthroline moiety of the TPPHZ ligand (Figure 47).

**Figure 48:**
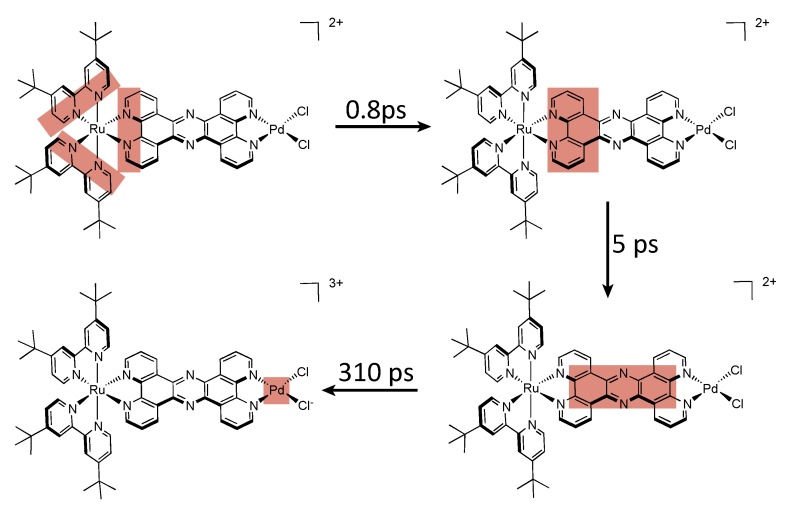
Charge distribution along the photophysical pathway for [Ru(tbbpy)_2_(TPPHZ)PdCl_2_]^2+^ [206].

This transfer occurs with a time constant of 1.2 ps for the mononuclear complex, and with a time constant of 0.8 ps for the bimetallic compound (Figure 48). Then, decay from this ^3^MLCT state towards an ILCT state localized on the phenazine moiety of TPPHZ, takes place. This process is drastically influenced by the presence of the palladium center. Indeed, this decay happens with a time constant of 240 ps for the mononuclear complex, while it proceeds with a time constant of 5 ps for the bimetallic entity. Finally, a LMCT occurs, that reduces the palladium center, with a time constant of 310 ps. 

**Figure 49:**
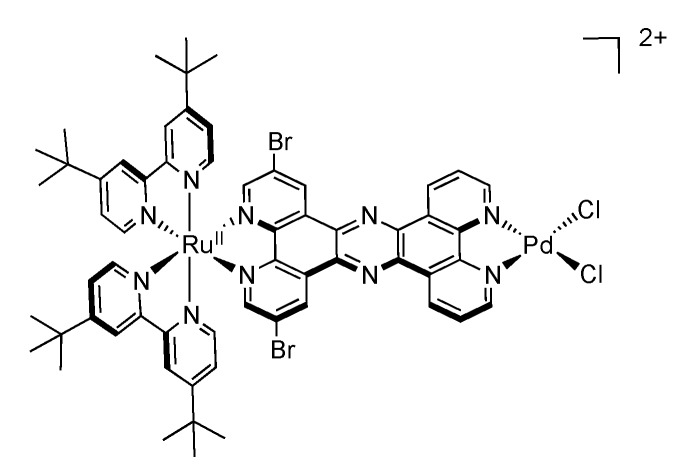
[Ru(tbbpy)_2_(TPPHZ)]^2+^ bearing a bromine substituted TPPHZ.

The TPPHZ ligand, central component of the system, plays different roles. Of course, it acts as a relay for the electron transfer and allows the interaction between the two metal centers, but it also has drastic influence on the photophysical scheme since it is involved in most of the deactivation processes. For example, the photocatalytic activity is controlled by the localization of the first excited state of the system. Since this excited ^1^MLCT state is delocalized on the tbbpy ligand as well as the TPPHZ ligand, it is of interest to try and populate only the TPPHZ ligand in the first ^1^MLCT state in order to design efficient photocatalysts [207]. For this purpose, TPPHZ was substituted by bromine atoms (Figure 49), and the photophysics of the resulting complex was studied [143,208]. 

Thus the first populated excited state is a MLCT state involving the phenanthroline part of TPPHZ, that is stabilized by the presence of the bromine substituents. This stabilization has nonetheless also an influence on the second step, *i.e.*, the decay towards the phenazine moiety, which becomes less favorable, going from 5 ps to 8 ps. Furthermore, the last process, *i.e.*, the LMCT is also lowered compared to the unsubstituted system, with a time constant going from 310 ps to 460 ps. Thus the introduction of bromine atoms leads to a deceleration of the different electron-transfer steps and flattens the electron-transfer gradient in a general way. Changing solvent from acetonitrile to a mixture acetonitrile/water 9:1, a mixture where the photocatalyst is active, does not lead to spectral changes, therefore the photophysical scheme described for the unsubstituted and bromine substituted TPPHZ complexes is still suitable. Nevertheless, the addition of water influences each electron-transfer process, especially the LMCT step. For example, the ILCT step that occurs with a time constant of 5 ps for the unsubstituted TPPHZ complex and 8.2 ps for the bromine substituted TPPHZ complex is decelerated to 6.1 ps and 10.5 ps respectively. While this decrease is not of great importance, the LMCT transition is decreased from 310 to 800 ps for the unsubstituted TPPHZ and from 460 to 850 ps for the bromine substituted TPPHZ. These results indicate that the electron-transfer gradient that was introduced by substituting TPPHZ with bromine atoms is highly compensated by solvent-induced effects. 

**Figure 50:**
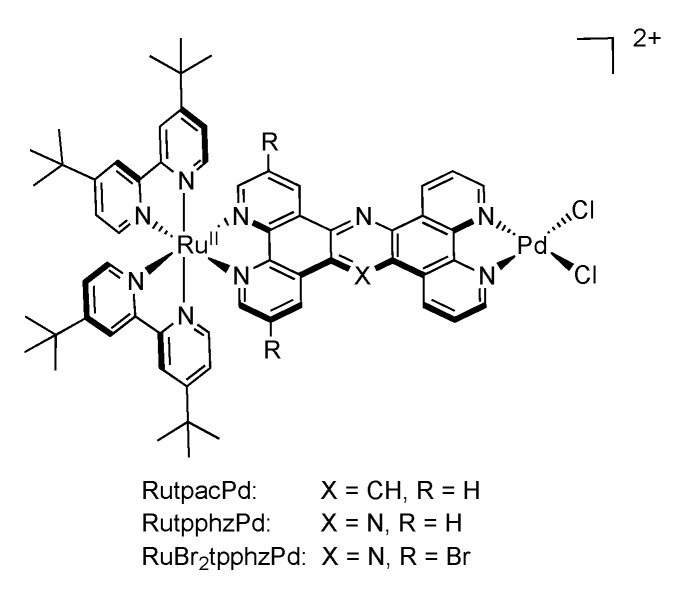
Ruthenium^II^ complex with various extended π-systems.

As the bridging ligand with extended aromaticity is a key component of the photocatalytic system, and knowing that water interacts with it and can thus affect particularly the LMCT transition, the skeleton of the ligand has also been changed (Figure 50, Table 13) [209].

Changing the phenazine moiety for an acridine moiety might in this frame disfavor the interaction between water molecules and the central core. Previous studies have indeed shown that complexes bearing a TPAC ligand, namely [Ru(phen)_2_(TPAC)]^2+^, have an excited state that is less sensitive towards water than related TPPHZ complexes. Two time constants, a first one that is of 4.4 ps in acetonitrile, and a second one of 580 ps can describe the photophysics of [Ru(tbbpy)_2_(TPAC)PdCl_2_]^2+^. The long component can be assigned, by comparison with the previously described complexes, to the LMCT from the acridine core towards the palladium center. Therefore, the first short component must include two steps, *i.e.*, the charge transfer from the ^1^MLCT towards the ^3^MLCT located on the phenanthroline moiety, and the ILCT from the phenanthroline moiety towards the acridine core. Once again, when water is added (10% in acetonitrile), the first time constant is not very much affected. The second time constant however is accelerated (340 ps instead of 580 ps), meaning that the LMCT from the acridine core towards the palladium center is favored. This acceleration is in agreement with the fact that water molecules interact mainly with the palladium center, probably by chloride substitution, and that substituting a nitrogen atom in the TPPHZ ligand by a CH group has only limited influence in the overall process, the most important moiety being the palladium catalytic center and its interaction with water. 

**Table 13:** Photophysical and catalytic data for representative ruthenium^II^ complexes. Lifetimes are measured in aerated acetonitrile [209].

Complex	λ_abs_/nm	λ_em_/nm	τ/ns	Solvent (catalysis)	Donor	TON (time (h))
[Ru(tbbpy)_2_(TPAC)PdCl_2_]^2+^	475	617	180	ACN + 10% H_2_O	TEA	138.7 (18)
[Ru(tbbpy)_2_(TPPHZ)PdCl_2_]^2+^	445	650	27	ACN + 15% H_2_O	TEA	238.3 (18)
[Ru(tbbpy)_2_(Br_2_TPPHZ)PdCl_2_]^2+^	484	675	84	ACN + 7.1% H_2_O	TEA	94.2 (18)

**Figure 51:**
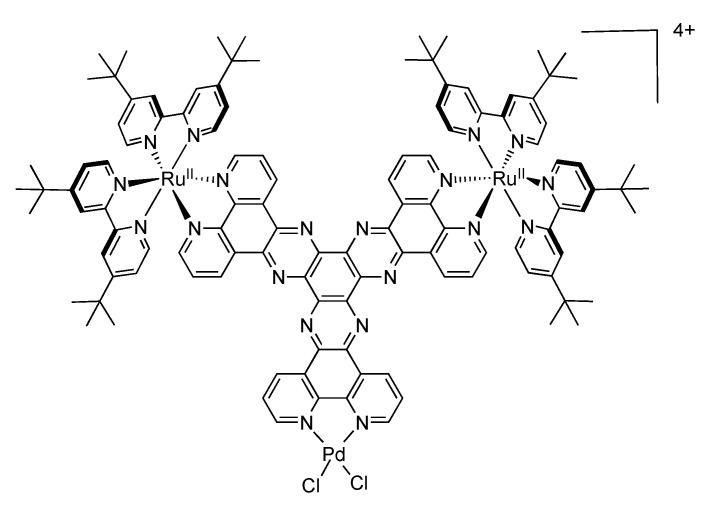
{[(Ru(tbbpy)_2_)_2_(µ-PHAT)]PdCl_2_}^4+^.

It is interesting to mention the experiments realized on the PHAT ligand (PHAT = phenanthroline-HAT = 9,10,19,20,29,30-hexaazahexapyrido[3,2-a:2',3'-c:3'',2''-k:2''',3'''-m:3'''',2''''-u:2''''',3'''''-x]-tri-naphthylene). This ligand, first synthesized by Lehn and coworkers [210], presents the particularity of having a HAT central core, linked to three phenanthroline units. Using this ligand, Rau and coworkers synthesized a heteronuclear complex, presenting two ruthenium centers, and a PdCl_2_ unit, in view of designing a system capable of hydrogen production (Figure 51) [211].

Unfortunately, although this system looked promising, it did not produce hydrogen. A possible explanation could be that the electron would be transferred from the Ru^II^ chromophores towards the PHAT ligand, that would not be localized on the phenazine-type moiety, but rather delocalized over the whole π-accepting HAT core. This delocalization would lower the driving force for reduction of the palladium center, hence would induce the complete loss of activity. 

### 5.2. Development of Ligands with More Extended π-Systems

**Figure 52:**
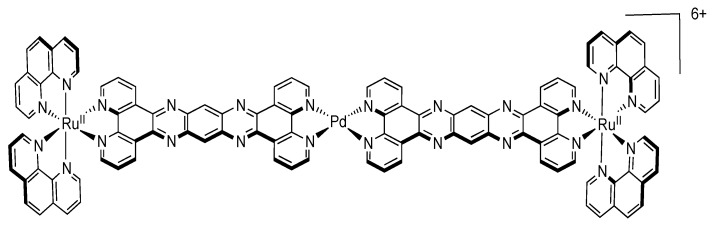
The trinuclear complex based on tatpp [Pd{(tatpp)Ru(phen)_2_}_2_]^6+^.

The synthesis of bimetallic ruthenium complexes having a planar, rigid and aromatic bridging ligand has been an active research area since mid 1990s. These new molecular platforms were developed as dendrimers, photo-catalysts or molecular wires. In this context Lehn and coworkers first synthesized a trinuclear complex based on tatpp (tatpp = 9,11,20,22-tetraazatetrapyrido[3,2-a:2',3'-c:3'',2''-l:2''',3'''-n]pentacene) [126] (Figure 52).

**Figure 53:**
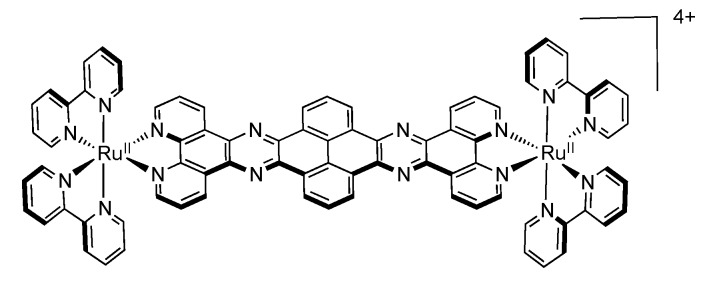
[Ru(bpy)_2_(bqpy)Ru(bpy)_2_]^4+^.

Later on, Launay’s group prepared a binuclear Ru^II^ complex based on bis{dipyrido[3,2-f:2',3'-h]quinoxalo}[2,3-e:2',3'-l]pyrene (bqpy) (Figure 53) that, due to its central pyrene core, is able to induce supramolecular π-π dimerization [212].

**Scheme 1:**
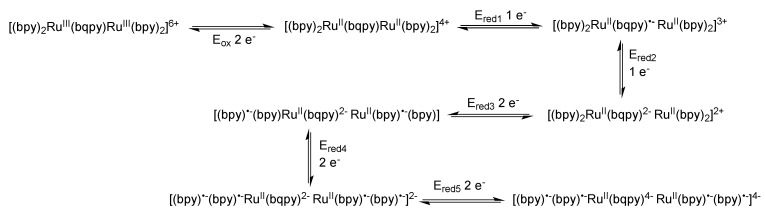
Redox pathway for [Ru(bpy)_2_(bqpy)Ru(bpy)_2_]^4+^.

Despite its extended aromatic character, this luminescent complex that has a size of 20 Å, can unfortunately only be seen as four non-interacting parts, as the distance between the two metal centers precludes strong coulombic interaction [213]. Indeed, it can be seen as two separated [Ru(bpy)_3_]^2+^ and two phenazine moieties. The first oxidation occurs as a two-electron single wave at +1.35 V *vs.* SCE and involves each ruthenium center. Regarding the reduction, two one-electron processes occur at −0.9 V and −1.07 V *vs.* SCE respectively, that are anodically shifted as compared to the value for [Ru(bpy)_3_]^2+^ and attributed to the consecutive addition of two electrons on the bqpy. This is in agreement with the highest π-acceptor character of the bqpy ligand. Simultaneous reductions of the ancillary bipyridine ligands can be achieved at −1.31 V and −1.52 V *vs.* SCE, in agreement with the reduction potential of [Ru(bpy)_3_]^2+^. Finally, a last reduction wave that involves two electrons, probably two very close monoelectronic processes, can be observed at −1.86 V *vs.* SCE, corresponding to further reduction of the bqpy ligand (Scheme 1). 

This binuclear complex can also be seen as two [Ru(bpy)_2_(TPPHZ)]^2+^ separated by a pyrene core, as it shows indeed similarities with [Ru(bpy)_2_(TPPHZ)]^2+^, with almost the same emission energy, lifetime and quantum yield. 

### 5.3. Complexes based on Ligands with Extended Aromaticity for Electron Photo-Accumulation

In order to design systems capable of storing several electrons and elaborating efficient artificial photosynthetic systems, MacDonnell *et al.* developed two binuclear ruthenium complexes, [Ru(phen)_2_(tatpq)Ru(phen)_2_]^4+^ and [Ru(phen)_2_(tatpp)Ru(phen)_2_]^4+^ [129]. The former is able to photo-accumulate up to four electrons and four protons on the central bridging ligand [63,214]. This complex presents a bielectronic oxidation at +1.37V *vs.* SCE, indicating poor interaction between the two metal centers. Interestingly, it exhibits a reversible mono-electronic reduction wave and a quasi-reversible wave at −0.23 V and −0.60 V respectively (Table 14). Comparing these reduction values with the ones of the related [Ru(phen)_2_(TPPHZ)Ru(phen)_2_]^4+^, it could be estimated that the driving force for the electron transfer from a Ru^II^ core towards the quinone part of the bridging ligand is favored by −0.55 eV. This enhanced driving force can explain the fact that [Ru(phen)_2_(tatpq)Ru(phen)_2_]^4+^ is non-luminescent in acetonitrile, even at 77 K in frozen matrix. This phenomenon was indeed attributed to an intramolecular electron transfer to specific part of the bridging ligand. 

**Table 14:** Redox potentials *vs.* SCE for [Ru(phen)_2_(tatpp)Ru(phen)_2_]^4+^ and [Ru(phen)_2_(tatpq)Ru(phen)_2_]^4+^ measured in acetonitrile. In parenthesis is the number of exchanged electrons.

Complex	Oxidation	Reduction
[Ru(phen)_2_(tatpp)Ru(phen)_2_]^4+^ [161]	+1.32 (2)	−0.26 −0.75 −1.32 (2)
[Ru(phen)_2_(tatpq)Ru(phen)_2_]^4+^ [129]	+1.37 (2)	−0.23 −0.60

**Figure 54:**
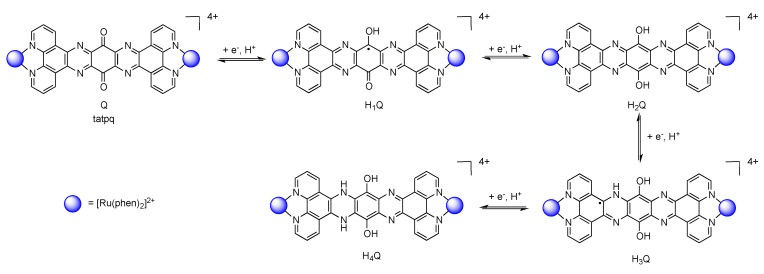
Representation of the related redox and protonation isomers for [Ru(phen)_2_(tatpq)Ru(phen)_2_]^4+^.

Light excitation induces an electron transfer from the Ru^II^ to the bridging ligand. Then triethylamine or triethanolamine used as a sacrificial donor, reduces the Ru^III^ center, which regenerates the initial Ru^II^ ion. Next the reduced tatpq protonates, which produces a singly reduced neutral system. A second reduction subsequently takes place on this system. This sequence of reduction, protonation can be repeated four times until four electron and four protons are accumulated in the reduced bridging ligand (Figure 54). Thus this system, in the presence of a sacrificial donor and in deoxygenated solution, acts as a multielectron collector. 

The related compound, *i.e.*, [Ru(phen)_2_(tatpp)Ru(phen)_2_]^4+^ also presents a two-electron oxidation wave at +1.32 V *vs.* SCE, meaning that the two Ru^II^ chromophores act as two separated chromophores, so that the complex can be considered as two [Ru(phen)_3_]^2+^ chromophores and a central tetraazapentacene acceptor unit. When tatpp ligand is used, two reversible one-electron reduction waves are observed at −0.22 V and −0.75 V *vs.* SCE. Once again, an increase in the driving force for the electron transfer by −0.52 eV, compared to [Ru(phen)_2_(TPPHZ)Ru(phen)_2_]^4+^, can be held responsible for the lack of emission in acetonitrile or in frozen matrix. 

**Figure 55:**
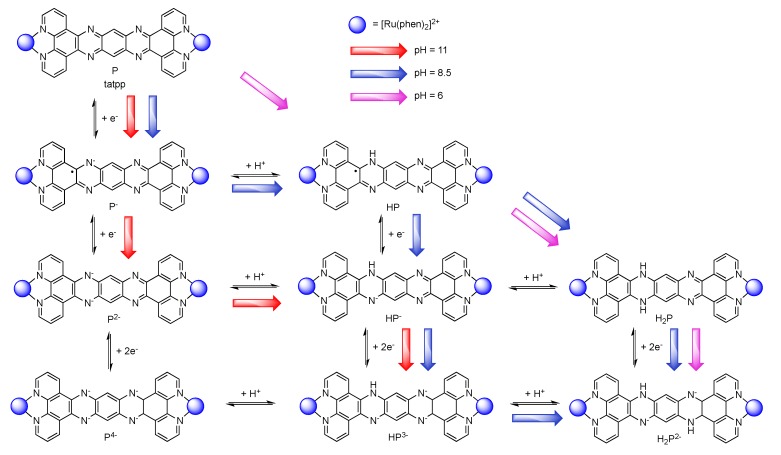
Electron and proton transfer processes in water at pH 11 (red), 8.5 (blue) and 6 (pink) [216].

[Ru(phen)_2_(tatpp)Ru(phen)_2_]^4+^ was shown to be able to accept up to four electrons and two protons on its central ligand [215,216]. It is important to notice that, although electroreduction is a more complex study in water than in acetonitrile, this behavior is maintained in a mixture constituted of water and acetonitrile. The absorption spectra of these two reduced species depend on the solution pH, indicating therefore possible protonation mechanism at the reduction site (Figure 55). 

Up to seven different species of [Ru(phen)_2_(tatpp)Ru(phen)_2_]^4+^ could be identified using spectroelectrochemistry and photochemistry, all of them being interconnected by electron transfer and protonation/deprotonation processes. The proposed mechanism for the two reductions depends strongly on pH. Indeed, when the pH is superior to 11, sequential reduction of P to P^−^ and then to P^2−^ is favored. On the contrary, at lower pH a proton-coupled bielectronic process is favored. For pH 11 and 8.5, it is clear that sequential one electron and proton-coupled one-electron processes are observed, but these processes all merge at pH 7 or below.

**Figure 56:**
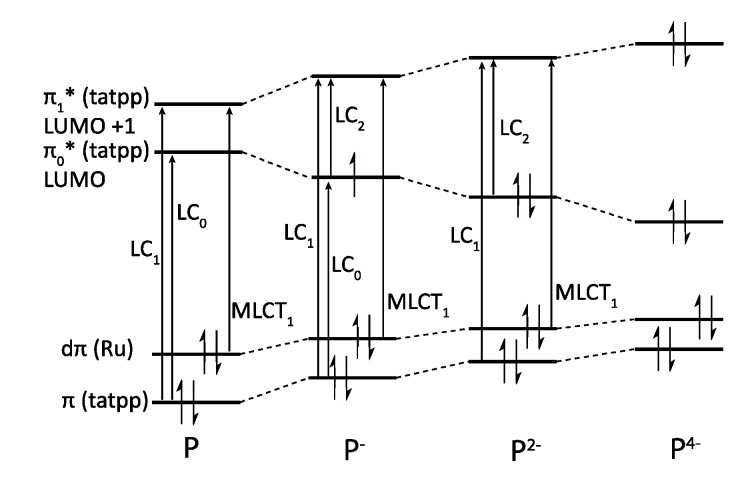
Qualitative molecular orbital energy diagram for the redox and protonation isomers of [Ru(phen)_2_(tatpp)Ru(phen)_2_]^4+^ [215].

The proposed molecular energy diagram for [Ru(phen)_2_(tatpp)Ru(phen)_2_]^4+^ and its photo-reduced derivatives is presented in Figure 56.

Interpreting the qualitative molecular orbital energy diagram is a key feature to the understanding of the orbitals that are involved in the most important steps. Interestingly, π_1_* can be seen as a LUMO that is similar to phenanthroline ligand at both ends of the bridging ligand. This orbital is the one that is populated when the MLCT band is photoexcited. This transition Ru(dπ) – tatpp(π_1_*) is responsible for the broad absorption spectrum centered between 440 and 480 nm. Further transitions, at 325 nm and 445 nm are assigned to transitions centered on the tatpp ligand itself, being respectively a π-π_1_* (LC_1_) and a π-π_0_* (LC_0_) transition. It is worth mentioning that the transition between the Ru(dπ) orbital and the π_0_* orbital is not observed, due to poor electronic coupling between these two orbitals [152,155,217]. When [Ru(phen)_2_(tatpp)Ru(phen)_2_]^4+^ is reduced in P^−^, the π_0_* is populated, which results in the stabilization of its energy level. A new ligand centered transition, lower in energy is therefore possible. This new LC_2_ transition appears as two bands, mainly because of vibronic fine structure, that are centered at 855 and 965 nm. Reducing P^−^ to P^2−^ populates entirely the π_0_* orbital, lowering once again its energy and destabilizing slightly the π_1_*, thus the previously LC_2_ transition is now blue-shifted to 635 nm and 685 nm. Finally, complete bleaching of the LC transition is observed upon further reduction to P^4−^. 


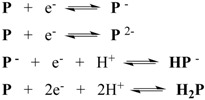


Thus, in [Ru(phen)_2_(tatpp)Ru(phen)_2_]^4+^, the central core can be seen as the acceptor species, and each peripheral [Ru(phen)_2_]^2+^ fragment constitutes an antenna subunit. These three components are only weakly coupled in the binuclear species, thus in the bridging tatpp, the terminal acceptor orbital is only weakly coupled to the ruthenium dπ orbitals. Even if this orbital is the one responsible for electron storage, it is not the orbital that is populated upon photo-excitation. Furthermore, there are multiple reduction states that can be reached at potentials that are more positive than those for the terminal phenanthroline ligand. Finally, nitrogen atoms from the tetraazaanthracene moiety are easily accessible for protonation

## 6. Conclusions

Although the photophysical model for [Ru(bpy)_3_]^2+^ is considered as the prototypical model for the vast majority of ruthenium^II^ polypyridyl complexes, there are numerous ruthenium^II^ complexes with an extended aromatic π-system whose photophysical scheme differs from the classical one. It has been necessary over the time to introduce various excited states, bright, dark, ligand-centered to take into account the different deactivation pathways that were observed. We have discussed in this review the different aspects that rule the photophysics of such ruthenium^II^ complexes. We have also briefly reviewed the energy and/or electron transfer that occur between different metal centers linked by a bridging ligand, emphasizing mainly the communication between the different metal centers, as well as on the influence of the solvent on the different deactivation pathways. The potential use of these π-extended bridging ligands in photo-catalysis for hydrogen production as well as their ability to photo-store electrons and protons has been discussed. There are of course numerous systems useful for hydrogen production and water splitting that were only cited in this review, but that deserve a lot of attention as many of these systems could pave the way for the development of efficient photo-catalysts that shall meet the growing energy demand. 
